# Single genome retrieval of context-dependent variability in mutation rates for human germline

**DOI:** 10.1186/s12864-016-3440-5

**Published:** 2017-01-13

**Authors:** Aleksandr B. Sahakyan, Shankar Balasubramanian

**Affiliations:** 1 Department of Chemistry, University of Cambridge, Lensfield Road, Cambridge, CB2 1EW UK; 2 Cancer Research UK Cambridge Institute, University of Cambridge, Li Ka Shing Centre, Robinson Way, Cambridge, CB2 0RE UK; 3 School of Clinical Medicine, University of Cambridge, Cambridge, CB2 0SP UK

**Keywords:** Nucleotide substitutions, Spontaneous mutations, Germline, Context-dependence, Genome composition, Somatic mutations, Cancer

## Abstract

**Background:**

Accurate knowledge of the core components of substitution rates is of vital importance to understand genome evolution and dynamics. By performing a single-genome and direct analysis of 39,894 retrotransposon remnants, we reveal sequence context-dependent germline nucleotide substitution rates for the human genome.

**Results:**

The rates are characterised through rate constants in a time-domain, and are made available through a dedicated program (Trek) and a stand-alone database. Due to the nature of the method design and the imposed stringency criteria, we expect our rate constants to be good estimates for the rates of spontaneous mutations. Benefiting from such data, we study the short-range nucleotide (up to 7-mer) organisation and the germline basal substitution propensity (BSP) profile of the human genome; characterise novel, CpG-independent, substitution prone and resistant motifs; confirm a decreased tendency of moieties with low BSP to undergo somatic mutations in a number of cancer types; and, produce a Trek-based estimate of the overall mutation rate in human.

**Conclusions:**

The extended set of rate constants we report may enrich our resources and help advance our understanding of genome dynamics and evolution, with possible implications for the role of spontaneous mutations in the emergence of pathological genotypes and neutral evolution of proteomes.

**Electronic supplementary material:**

The online version of this article (doi:10.1186/s12864-016-3440-5) contains supplementary material, which is available to authorized users.

## Background

The stability, organisation and dynamics of genomes are key factors that influence the molecular evolution of life [1]. Genomic single-nucleotide mutations, with their subsequent fixation in a population, occur an order of magnitude more frequently than common insertions/deletions [2, 3], hence are major contributors in defining the genome evolution. An understanding of the descriptors that govern single-nucleotide mutations and substitutions is thus essential to comprehend genome dynamics and its link to the underlying molecular processes.

For the sake of lucidity in pointing to different contributions on substitution rates, which may encompass both mutation- and fixation-based effects, let us introduce a break down of the rate into a number of general components. For a given genomic position and *i*→*j* nucleotide conversion, the substitution rate, as expressed by the rate constant *r*_*i*,*j*_, can be roughly presented as a single-base average value $r_{i,j}^{sb}$ and fluctuations contributed by short-range context ($\delta r_{i,j}^{sr}$), CpG-associated ($\delta r_{i,j}^{CpG}$), long-range ($\delta r_{i,j}^{lr}$), gene/functional ($\delta r_{i,j}^{gene}$), and specific ($\delta r_{i,j}^{spec}$) effects (1):

1 $$ r_{i,j} = r_{i,j}^{sb} + \delta r_{i,j}^{sr} + \delta r_{i,j}^{CpG} + \delta r_{i,j}^{lr} + \delta r_{i,j}^{gene} + \delta r_{i,j}^{spec}   $$

The $r_{i,j}^{sb}$ term can be estimated through genomic averages for the individual *i*→*j* substitutions, and has been explored for the genomes of human [4, 5] and other species [6–8]. By investigating the aggregation patterns in substitution frequencies, it was shown that the *r*_*i*,*j*_ variation is subjected to two distinct, short-range (< 10 nt) and long-range (> 1000 nt), effects [9, 10]. In the equation above, the short-range effect is captured through the $\delta r_{i,j}^{sr}$ term and mainly describes the totality of the intrinsic properties and sequence-dependent interactions of DNA with overall mutagenic and reparation processes in a given organism [11]. The better-studied substitution patterns at a CpG context [12–14] are separated in the $\delta r_{i,j}^{CpG}$ term, since besides having a specific short-range dyad context, the CpG mutations underlying the $\delta r_{i,j}^{CpG}$ substitution term also depend on a number of regional factors that alter the epigenetic targeting of the CpG sites [10, 15–18]. Many relatively recent studies provide essential insights into the $\delta r_{i,j}^{lr}$ variation caused by the regional effects that depend on a long-range (megabase) sequence context through general mechanisms, such as recombination and GC-biased gene conversion [1, 19–21], transcription-coupled biased genome repair [22] and instability [23], chromatin-organisation- [24] and replication-associated mutational bias [25] and inhomogeneous repair [26], differential DNA mismatch repair [27], non-allelic gene conversion [28], and male mutation bias [29]. The term $\delta r_{i,j}^{gene}$ captures the change in substitution rates in genes and other functional elements under elevated selection bias and may reflect observations such as the increased neutral substitution rates in exons [30, 31] and the possible reduction of mutation rates in the X-chromosome [32]. $\delta r_{i,j}^{spec}$ holds the highly specific increase or decrease in substitution rates governed by a strong selection or targeted underlying hyper- and hypomutations [33] present, for example, in the genes of immune system, and may additionally include other effects not captured in prior terms.

Herein, we propose a methodology to obtain the core components (2, capturing only the short-range sequence caused variation) of the neutral single-nucleotide substitution rates *via* the direct analysis of 39,894 L1 mobile DNA remnants [34] in the same, human, genome (a single-genome approach).

2 $$ r_{i,j}^{core} = r_{i,j}^{sb} + \delta r_{i,j}^{sr}   $$

Our **tr**ansposon **e**xposed **k**-mer rate (Trek) method provides the $r_{i,j}^{core}$ rate constants at single-nucleotide resolution in L1, where we demonstrate sufficient sequence variability to cover a wide-range of sequence contexts. We use this coverage to determine the core rate constants for all possible nucleotide substitutions (3 per position) at each of the 3.2 billion positions in the human genome. The Trek aims at revealing the $r_{i,j}^{core}$ variation in a relatively model-free manner and at a level beyond accounting for only the two immediate neighbouring nucleotides [35]. We make our dataset of the time-dependent rate constants for individual substitutions publicly available. To exemplify the usage of Trek data, we demonstrate that the $r_{i,j}^{core}$ values can generate a sequence starting from a random DNA sequence, whose key features are in better agreement with the short-range oligomeric organisation of the human genome. We next calculate the basal substitution propensity profile of the human genome, evaluating the core predisposition to single-nucleotide substitutions. We outline the decreased frequency of the sequence motifs that are stable in germline among the sites linked to somatic cancer mutations.

## Results and discussion

### Revealing the core single-nucleotide substitution rates

**Fig. 1:**
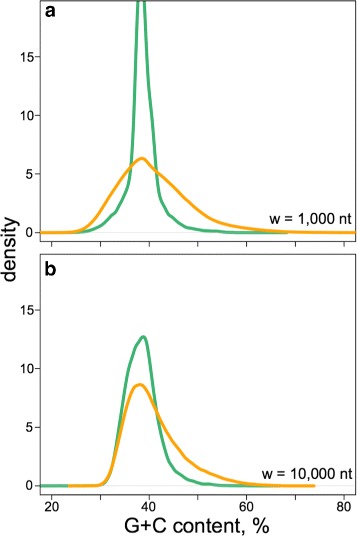
Long-range G+C context of the young L1 insertion sites in the human genome. **a** and **b** The distribution of the G+C contents for all the w-sized (1000-nt in **a** and 10,000-nt in **b**) bins in the human genome (*orange lines*) is shown, as compared to the same distribution but using only the bins centred at the midpoints of all the remnants of young (L1Hs, L1PA2, L1PA3, L1PA4, L1PA5) L1 elements (*green lines*)

The repetitive occurrence of mobile DNA elements in different regions within the same genome [34] provides the opportunity to obtain the $r_{i,j}^{core}$ (2) rate constants that account for the $\delta r_{i,j}^{sr}$ immediate effects of neighbouring nucleotides. After the initial inactivation at different time epochs [36–39], individual remnants of many transposon subfamilies within a genome have been subjected to largely the same overall mutagenic and repair conditions as the rest of the genome [40], hence can also serve as markers of $r_{i,j}^{core}$ neutral substitution rates applicable to genomic sites that share the immediate sequence-context. For the purpose of this study, we have used the hominoid lineage of the L1 (long interspersed nuclear element 1, LINE-1) retrotransposons, spanning 3.1 to 20.4 myr (million years) of age [37]. The constituent subfamilies of the lineage are L1PA5, L1PA4, L1PA3, L1PA2 and the most recent L1Hs. Their respective age and the number of insertions in the human genome are presented in Table S1 in Additional file 1. The choice was made through the following reasoning. The L1 elements have a long (∼6 k nt) sequence without extended repeats like in the LTR (long terminal repeat) elements [34]. This enables their robust mapping on a chosen template and provides essential local sequence variability around different nucleotide positions within L1 elements. There are distinct L1 subfamilies that were active at different time epochs, with detailed molecular clock analyses available [36–39] to reveal and, importantly, validate the age of each subfamily. They are well-represented and, unlike other classes of transposable elements, are uniformly scattered across mostly the intergenic regions of the human genome [34, 41, 42], and are less prone to recombination and context bias [43, 44]. The young L1 subfamilies have most of their remnants coming from the genomic regions with G+C content close to the genomic average value (Fig. 1, see also [41]). Unlike SINEs (small interspersed nuclear elements) and LTRs, LINE sites show a very low level of RNA polymerase enrichment, as a marker of transcriptional association, in normal tissues [45]. The selected most-recent subfamilies are sufficiently young [37] a) to enable an unambiguous identification of the genomic coordinates of the borders for the remnants; b) to assume that each position in those elements would be unlikely to undergo repeated substitutions over the studied period of their existence as remnants in the human genome (see Methods); c) to attribute a time-invariance to the rates during the analysed period of mutation accumulation [20, 46, 47]. Finally, many matching positions in our studied five L1 representatives share the same consensus bases, hence, such positions are not polymorphic due to adaptive pressure and can serve as internal references for inferring the $r_{i,j}^{core}$ rates.

**Fig. 2:**
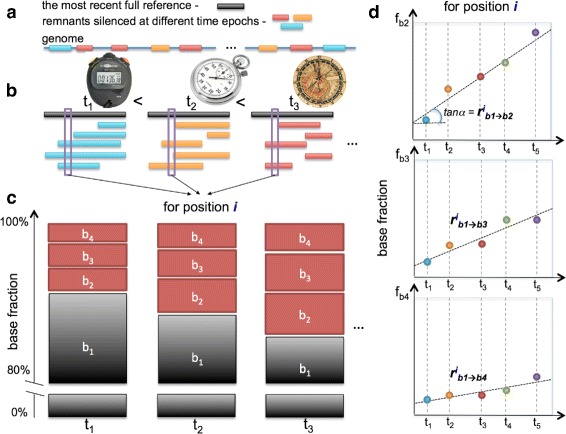
Single genome determination of the context-dependent substitution rate constants. **a**–**d** The Trek approach is applicable to a genome containing multiple remnants of retrotransposon subfamilies silenced at different time epochs (**a**). We can consider those subfamilies as substitution counters that had different resetting ages (**b**). The full consensus sequence of the most recent subfamily is taken as a reference (**a**). The remnants are then grouped by their age and fully mapped onto the reference sequence (**b**). For each position *i* in the reference sequence, the fractions of the four bases in all the time groups are calculated (**c**). The comparison of these fractions coming from individual base types across different time periods enables a linear model fitting, through which we can reveal the rates for the substitutions into the *b*_2_, *b*_3_ and *b*_4_ bases from the consensus (*b*_1_) state of the given position (**d**). The steps (**c**) and (**d**) are repeated for all the positions in the reference sequence, producing single-nucleotide resolution core substitution rate constants with sequence-context dependency as sampled in the reference sequence of the mobile element. To assure the high quality and neutrality of the retrieved rates, we accounted for the sites in the reference sequence that had at least 700 mapped occurrences in each time group (**b**), with the same wild-type variant being always the prevalent one (more than 80%) in each subfamily (**c**) and producing a Pearson’s correlation coefficient of at least 0.7 in the time-evolution plots (**d**)

**Fig. 3:**
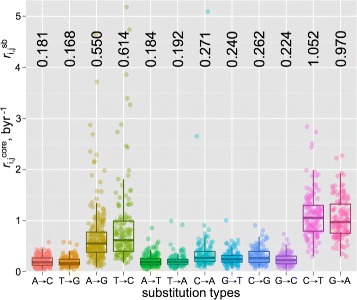
Transposon exposed (Trek) $r_{i,j}^{core}$ substitution rate constants of the human genome. The boxplots are shown for each *i*→*j* substitution type inferred from the hominoid L1 remnants spread across the human genome. Each point comes from a specific position in the L1 element, reflecting the substitution rate constant averaged across multiple occurrences of that specific position with the same sequence-context in multiple regions of the human genome. The complementary *i*→*j* pairs are plotted in adjacency. The median values of the overall substitution rates ($r_{i,j}^{sb}$) in byr ^−1^ (billion years) unit, averaged across the varying sequence-context within the L1 elements, are shown on the top

The Trek methodology of obtaining $r_{i,j}^{core}$ rates, along with the considerations for filtering out the possible selection and non-neutral substitution sites, is presented in Fig. 2 with further details in Methods, Figures S1 and S2 in Additional file 1. The acquired data on the full set of position-specific substitution rates are presented in Fig. 3, to highlight the revealed variation per substitution type, along with the fully averaged values for the rate constants.

A total of 661 positions, at the 3’ side of the L1 elements, passed our robustness checks (see Methods) and were thus employed to infer the corresponding $r_{i,j}^{core}$ values from the analysis of all the young L1 remnants in the human genome. We recorded the data in the Trek database that contains a set of well-defined $r_{i,j}^{core}$ constants (see below for the extent of sequence context coverage in the Trek database) capturing the influence of the unique arrangement of neighbouring nucleotides at those positions. Owing to the nature of the selected L1 elements, as discussed above, and the Trek procedure design (Fig. 2), we expect the absence of the $\delta r_{i,j}^{gene}$ contribution, the elimination of $\delta r_{i,j}^{lr}$ at the averaging stage (Fig. 2 b) and the removal of the $\delta r_{i,j}^{CpG}$ and $\delta r_{i,j}^{spec}$ effects through our robustness checks embedded within the Trek procedure (see Fig. 2 c, d and Methods). Therefore, our method provides the $r_{i,j}^{core}$=$r_{i,j}^{sb}+\delta r_{i,j}^{sr}$ core variation (Fig. 3) of the substitution rates at around the $r_{i,j}^{sb}$ genomic average values for each *i*→*j* base substitution. If the above is correct and Trek indeed results in $r_{i,j}^{core}$ values, further averaging of the core $r_{i,j}^{sb} + \delta r_{i,j}^{sr}$ rates (median values shown in Fig. 3) should give us the single-base $r_{i,j}^{sb}$ genomic average substitution rates, cancelling out the remaining $\delta r_{i,j}^{sr}$ contribution. In fact, the comparisons of our Trek-derived $r_{i,j}^{sb}$ with two published datasets that reflect on the genomic average $r_{i,j}^{sb}$ rates [4, 20] show an excellent correlation (Figure S3 in Additional file 1, Pearson’s R > 0.99) confirming the absence of any biased averaging and unusual substitution rates in the time-accumulated substitutions at the L1 sites that pass the Trek procedure. The genome simulation, described later in this work, provides an additional validation for our rate constants. The $r_{i,j}^{core}$ values (Eqs. 1 and 2) for all possible *i*→*j* neutral substitutions inferred for each of the eligible individual L1 positions are thus assumed to be common for any other sites in the human genome that share the short-range sequence context.

### The influence range of neighbour nucleotides

To apply the $r_{i,j}^{core}$ constants to the human genome, we first established the optimal length of a DNA sequence (k-mer, where k is the length of the sequence) capturing most of the influences that modulate substitution rates of the base at the centre. For this, we evaluated the power of the knowledge of the neighbouring arrangement of nucleotides in predicting the $r_{i,j}^{core}$ constants for each of the twelve *i*→*j* substitution types, where *i* and *j* are the four DNA bases. We built test predictors for individual substitution types *via* a tree-based gradient boosting machine (GBM, machine learning technique) [48, 49], while using varying lengths of sequences centred at the positions where the rate constants were to be predicted (see Methods). The aim of the machine learning procedure was to establish the optimal sequence length to minimise the error in the predicted rate constants (Figures S4 and S5 in Additional file 1). In agreement with prior evidence [9, 10, 44, 50], but now obtained for each individual *i*→*j* substitution type from Trek data, the optimal window was found to be 5-7-nt (both 5- and 7-nt resulting in comparable results for many substitution types) and was subsequently used as guidance for the direct mapping of the Trek rate constants from the L1 sequence onto any given human nuclear DNA sequence for the $r_{i,j}^{core}$ assignment.

### Mapping the Trek $r_{i,j}^{core}$ data on any DNA sequence

The upper 7-nt size window for determining the single-nucleotide substitution rate constants at the central base accounts for three upstream and three downstream bases relative to each nucleotide position. Our neutral substitution positions that pass the Trek criteria capture 636 unique 7-mers out of the possible 16,384 (4^7^). Therefore, for many loci in the human genome we need to use a smaller window (< 7-mer) as a match criterion to assign to one of the Trek rate constant sets. By trimming the size of the k-mer to five, hence accounting for two upstream and two downstream bases, we cover 404 unique sequences out of possible 1024 (4^5^). Further reduction of the size to three, allows having data for 56 unique triads out of 64 (leaving out only the CpG containing triads, see below). For the single-base case (1-mers), where we average out all short-range neighbour effects and longer-range sequence variability, we obtain data for all the four bases and 4×3 possible substitutions as shown in Fig. 3 (the median values on the top of the figure). The coverage of the longer k-mers is, however, increased nearly twice when we account for the strand-symmetry, as described in Methods. Please note, that for each unique k-mer we obtained three $r_{i,j}^{core}$ constants *via* the described analysis of a large pool of L1 remnants from different genomic loci.

With the above considerations, we created a program (Trek mapper, Methods, Note S1 in Additional file 1) to produce $r_{i,j}^{core}$ core substitution rate constants for any sequence, accounting for the context information within up to the 7-mer window and pulling the matching core data from the Trek database. Should a representative match be absent with the full 7-nt long sequence, the window around the given position in a query sequence is shortened into the longest variant possible (out of the 5-nt, 3-nt or 1-nt lengths) with a full match in the Trek database (Methods, Figure S6 in Additional file 1). In this way, for all the possible 16,384 7-mers, our Trek database reports 49,152 rate constants (3 ×16,384), of which 3168 (6.4%) account for the 7-mer context, 23,232 (47.3%) account for the nested 5-mer context, 17,120 (34.8%) for 3-mer and only 5632 (11.5%, CpG containing sequences) constants do not account for any context effect on the central base (since we eliminate those by design, due to the $\delta r_{i,j}^{CpG}$ contribution). Our full dataset reports and makes publicly available (Additional file 2), the time-dependent $r_{i,j}^{core}$ rates for all individual *i*→*j* substitutions accounting for the context effects beyond the 64 triads [35]. If we consider only the unique values in the Trek database, we report 2078 unique rate constants (taking into account different extent of averaging, where multiple entries are present for the different context ranges), of which 1208 (58.1%), 782 (37.6%), 85 (4.1%) and 3 (0.1%) entries account for 7-, 5-, 3- and 1-mer contexts respectively. The 1-mer averaged data were used for only the k-mers that contain either C or G bases of a CpG dyad at the centre, to assign the overall substitution rate constants by the Trek mapper. This was done since none of the CpG sites in the L1 remnants passed our robustness checks (Methods), due to the targeted, epigenetic- $(\delta r_{i,j}^{CpG})$ or APOBEC-affected single-nucleotide substitutions there [12–14]. The latter effects were likely to be non-uniform with time (active targeting, $\delta r_{i,j}^{spec}$, while in the viable epoch for each L1 subfamily, at both DNA and RNA levels) and may had been present in order to silence the active retrotransposons.

Our current data are for the human nuclear genome. However, the general approach for obtaining $r_{i,j}^{core}$ constants is applicable to any organism where the genome contains a set of well-characterised and related young mobile elements silenced at different time epochs and without notable genomic context bias.

### $r_{i,j}^{core}$ rates and the oligomeric composition of the human genome

**Fig. 4:**
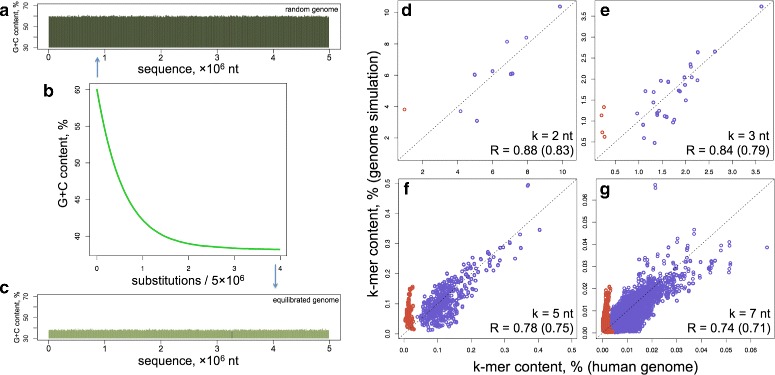
Comparison of the in silico evolved sequence and the actual human genome. **a** The 5-mln-nt starting sequence is randomly generated with 60% G+C content. (**b** and **c**) The sequence is then neutrally evolved using $r_{i,j}^{core}$ only, until the base-compositional equilibrium is established (**c**). This was reached after about 20 mln substitutions (or an average of 4 substitutions per site (**b**), where x-axis shows the number of substitutions divided by the simulated sequence length). The equilibration converges faster when we start from a sequence with lower G+C content. **d**–**g** The plots showing the correlation of the k-mer contents in the equilibrated genome with the corresponding content in the real human genome. The lengths of the k-mers along with the correlation coefficients are shown on the bottom right corners of the plots. Two correlation coefficients are shown with the exclusion and the inclusion (*the value in the bracket*) of CpG containing oligomers (*red points in the plots*). The *dashed lines* depict the diagonals for the ideal match of the k-mer contents

The full set of sequence-dependent human $r_{i,j}^{core}$ substitution rates (all three constants per position) enabled us to perform a sophisticated in silico evolution of a random DNA sequence, guided solely by our $r_{i,j}^{core}$ values. We started from a random sequence of 5 million (mln) nt with a G+C content of 60% (substantially greater than the 40.45% G+C content for the human genome). We performed random nucleotide substitutions weighted by Trek-inferred probabilities (Methods, Figure S7 in Additional file 1), where, after each cycle, the substitution rate constants were updated for the sequence positions that were either mutated or fell within the influence zone of the performed substitutions. The simulation was continued until the overall G+C content of the simulated sequence became constant (see Fig. 4 a–c).

The simulation converged to generate a sequence with the A, T, G and C compositions of 30.91, 30.90, 19.06 and 19.13% respectively. Note, that these values are close to the A, T, G and C compositions of the repeat-masked human genome of 29.75, 29.79, 20.24 and 20.22% respectively (Methods), being slightly AT rich. Furthermore, the simulated sequence captures the contents of different individual oligomers (k-mers) in the human genome. The data for all the possible 16 dyads, 64 triads, 1,024 pentads and 16,384 heptads are presented in Fig. 4 d–g and show a significant (see the correlation coefficients on the plots) correlation between the compositional landscapes of the Trek-simulated sequence and the actual human genome. Regardless of the starting composition of the initial DNA sequences, our simulations always equilibrated to a state with similar oligomer (up to 7-mer) content. The k-mer contents shown in Fig. 4 d–g for the actual human genome were calculated from the repeat-masked version of the RefSeq human genome, where all the identified repeat elements, including the L1, were disregarded. This assured the removal of a potential bias due to the presence of L1 elements (Methods), used to infer the rate constants, in the human genome. As $r_{i,j}^{core}$ constants are free of the $\delta r_{i,j}^{CpG}$ contribution (see above), the simulated genome produced higher alterations in representing the k-mer contents that have CpGs (red points in Fig. 4 d–g). These alterations visually demonstrate the role of $\delta r_{i,j}^{CpG}$ in the background compositional landscape of the human genome. The correlations in Fig. 4 are from simulations where the rate constants were symmetrised according to the inherent strand-symmetry in double-helical DNA (see Methods). The results without such equalisation are still significant, though producing slightly worse correlation coefficients (Figure S8 in Additional file 1).

**Fig. 5:**
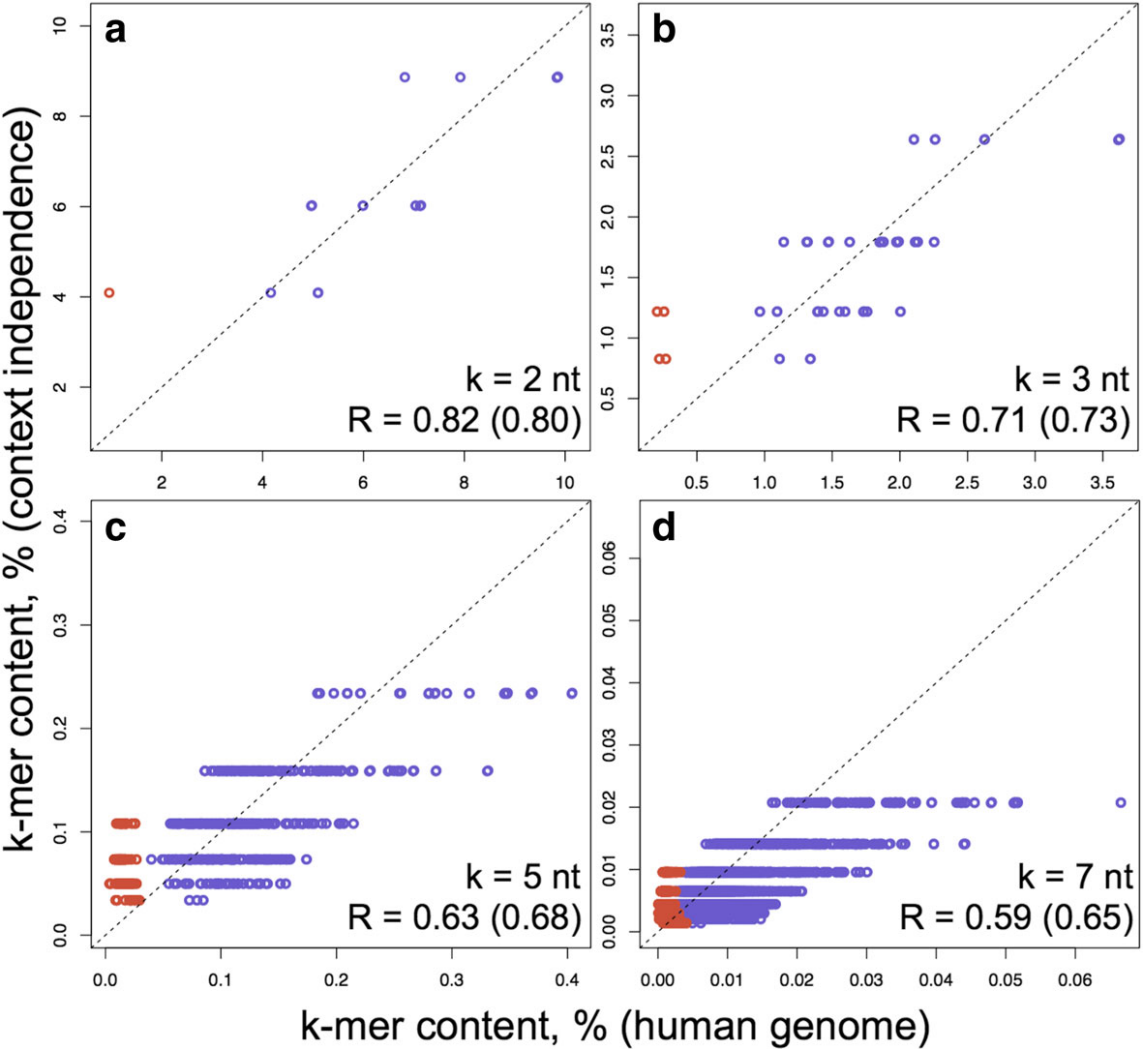
Oligomeric composition of the ideal neighbour-invariant sequence. **a**–**d** The oligomeric content of the human genome (*x-axis*) is compared to the content expected by chance (*y-axis*) in a sequence that has the exact single-base composition as the human genome, but has substitution rates that are purely context independent. This corresponds to a hypothetic genome simulation with perfectly correct $r_{i,j}^{sb}$ single-base rate constants, but without any $\delta r_{i,j}^{sr}$ sequence-context dependency present. The lengths of the k-mers, along with the Pearson’s correlation coefficients without and with (the values in the brackets) the CpG containing oligomer data (*red points*) are shown on the bottom right corners of the plots. The correlation coefficients are notably smaller compared to the in silico sequence, equilibrated based on the full set of context-dependent Trek $r_{i,j}^{core}$ constants. The *dashed lines* depict the diagonals for the ideal match of the k-mer contents

**Fig. 6:**
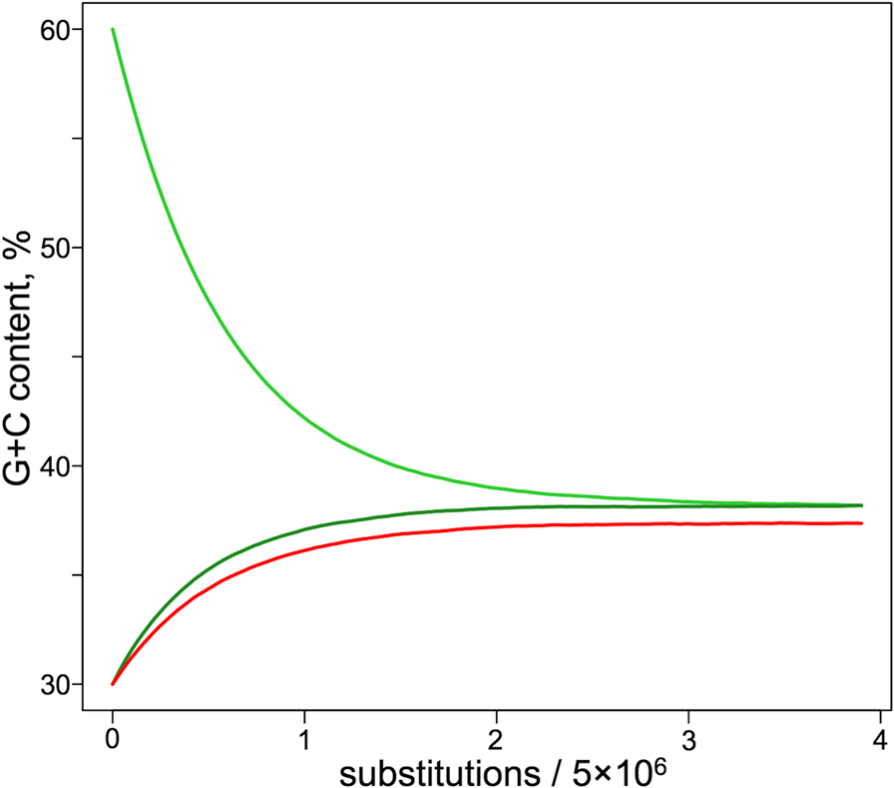
G+C content equilibration of random sequences with 60 and 30% initial G+C contents. Here, the in silico genome is equilibrated by using either the Trek $r_{i,j}^{core}$ constants with up-to 7-mer context dependence (*green lines*), or only the singleton rates from Trek without any context dependence (*red line*, shown for only the 30% G+C content start). The simulation accounting for the context dependence results in 38.19 and 38.18% of G+C content (compare to 40.45% for the repeat-masked human genome), starting from random sequences with 60% and 30% G+C contents respectively. The simulation with only the singleton rates converged at lower 37.36% value for the G+C content, where we expected the least disagreement between the “most-ideal” singleton vs. 7-mer description of the substitution rates (see Fig. 5 a). Please note, that in the hypothetic case of the most ideal singleton substitution rate constants, the discrepancy is more pronounced while analysing the genomic contents of the higher k-mers, as presented in Fig. 5

To confirm that the observed correlations for different k-mer contents (Fig. 4 d–g) present an improvement due to our sequence-context-dependent rates, rather than being a side effect, by a pure chance, in a sequence where the simulation makes only the single-base composition converge to that of the real human genome (such as in a sequence generated using an ideal 4×4 single-nucleotide substitution rate matrix), we calculated the expected distribution of different k-mers in a genome with fully random base arrangement but with the exact human A, T, G and C overall base composition. In the complete absence of any sequence-context effects, the probability of the occurrence (fraction) of any k-mer in a sufficiently long sequence is equal to the product of the occurrence probabilities of their constituent bases. For instance, the probability of observing the AGT triad is the *p*_*AGT*_=*p*_*A*_ *p*_*G*_ *p*_*T*_ product, where the individual *p*_*i*_ probabilities are the base contents expressed in fractions. The comparison of the k-mer fractions obtained in this way with the human genome data (Fig. 5) shows a substantially reduced correlation (for the genomic 7-mer content, Pearson’s R = 0.59 compared to 0.74 using Trek rates). The discrepancies are minimal while accounting for only the dyad and therefore singleton contents, however, while using context-invariant singleton substitution rates in the simplistic in silico simulations descried above, we observe slight but systematic underestimation of the overall G+C content in the sequence at compositional equilibrium (Fig. 6). Although excluded *via* the specificities of the Trek methodology (Fig. 2), to completely rule out the presence of any circularity in the reproduction of the human genome higher k-mer (up to 7 nt) content through the Trek rate constants, we also demonstrated the overall poor agreement between the k-mer contents of L1 elements used to infer the rate constants and the human nuclear genome (Figure S9 in Additional file 1).

The described simulations therefore support the attribution of a contributory role that the $r_{i,j}^{core}$ variability plays in shaping the compositional landscape of the human nuclear genome [51]. Importantly, our study demonstrates that the non-specific core substitution rates are capable of producing apparent selection or depletion patterns in higher k-mers, beyond dyads, in the human genome. To this end, the 7-mer content from our in silico equilibrated sequences, obtained solely based on the set of $r_{i,j}^{core}$ constants, can serve as a background standard to reveal specific selection [52] for or against different sequence motifs in the human genome.

Similar link between the context-dependent mutation pressure and the nucleotide composition has recently been shown for the triad counts in the bacterial genome of *Mesoplasma forum* [53] and in the human mitochondrial genome [54]. Interestingly, the latter study employed the somatic mutation propensities found by analysing cancer genomes to reproduce the triad count of the human mitochondrial genome, as an evidence of the link between the somatic and germline mutation rates in human mitochondria. That link is further visible in the cancer analysis presented here (*vide infra*) for the human nuclear genome.

### Basal substitution propensity profile of the human genome, substitution prone and resistant motifs

**Fig. 7:**
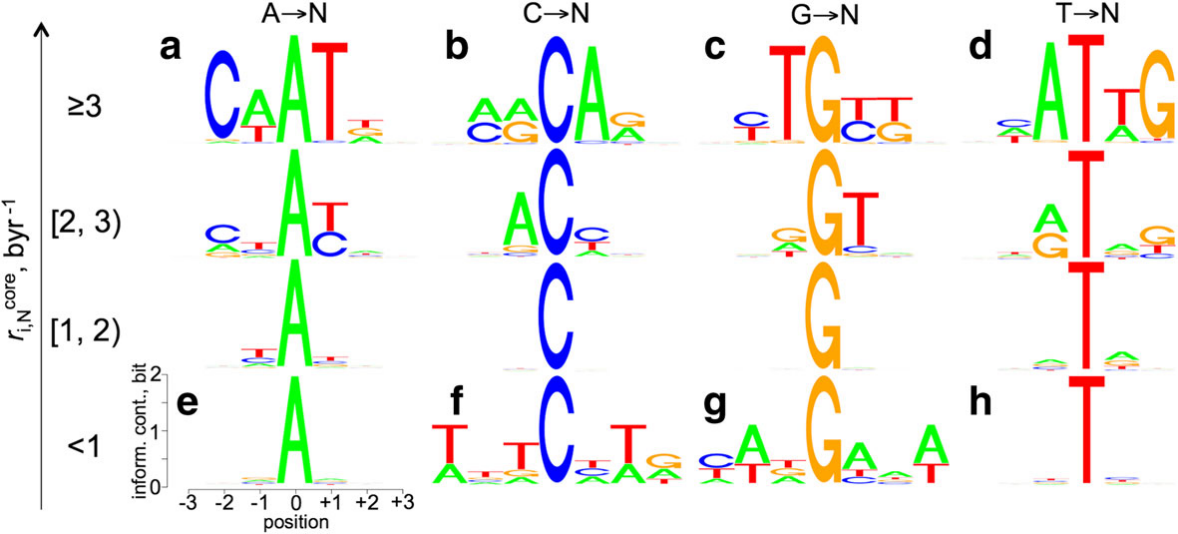
Sequence-context dependence of the $r_{i,N}^{core}$ basal substitution propensity (BSP) constants. **a**–**h** Sequence logos [55] are shown for all the unique 7-mer sequences grouped by the central base type (columns) and the category of the BSP range the sequences fall in (rows, BSP range is shown in byr ^−1^ rate constants). The *y-axes* in the individual sequence logos show the information content in bits. The *x-axes* outline the neighbouring base positions relative to the central base. For each sequence, the BSP of the central base (*i*) depicts the sum of the core rate constants for the substitutions to the three other (non-*i*) bases, $r_{i,N}^{core} = r_{i,b2}^{core} + r_{i,b3}^{core} + r_{i,b4}^{core}$. As can be seen from the plots, the bases A and T are highly mutable when the neighbouring positions are enriched in the same, A and T, bases (compare the logos **a** and **d** with **e** and **h**). The adjacent enrichment in A increases the BSP of C (**b**), and decreases the BSP of G (**g**) bases. Conversely, the adjacent enrichment in T increases the BSP of G (**c**) and decreases that of C (**f**) bases. Note, that our data are for $r_{i,N}^{core}$, thus independent from the methylation-driven increased mutation rates in CpG dyads [12–14]

Trek mapper provides the full set of $r_{i,j}^{core}$ constants for each position in the whole human genome. Such data enables us to calculate the germline context-dependent basal substitution propensity (BSP) by taking the sum of the individual rate constants for the three possible substitutions at each base position, thus producing the core $r_{i,N}^{core}$ constant for the substitution of a given base *i* by any other base *N*. Figure S10 in Additional file 1 shows the BSP profiles calculated for the individual chromosomes (red) as compared with the whole genome profile (green), where most of the chromosomes exhibit the same overall distribution as the whole genome. Further grouping and analysis [55] of the unique sequences found in regions of different BSP for the whole human genome reveals motifs with varying substitution propensities of the bases at the centre of 7-mers (Fig. 7). In particular, adhering to the standard nucleobase notation in small letters (a=A, c=C, g=G, k={G, T}, m={A, C}, n={A, C, G, T}, r={A, G}, t=T, w={A, T}, y={C, T}), the comparative examinations of the sequences reveal the overall stability of C in the *wntCnwn* context, and analogously, G in the *nwnGanw* context. Those bases become prone to substitutions, independently from the well-studied CpG context, in the *nmrCarn* and *nytGykn* motifs for the C and G bases respectively. Furthermore, A and T bases become prone to more frequent substitutions in the *ncwAtnn* and analogous *ngwTann* motifs (Fig. 7).

### Basal substitution propensity profile and cancer-linked somatic mutations

**Fig. 8:**
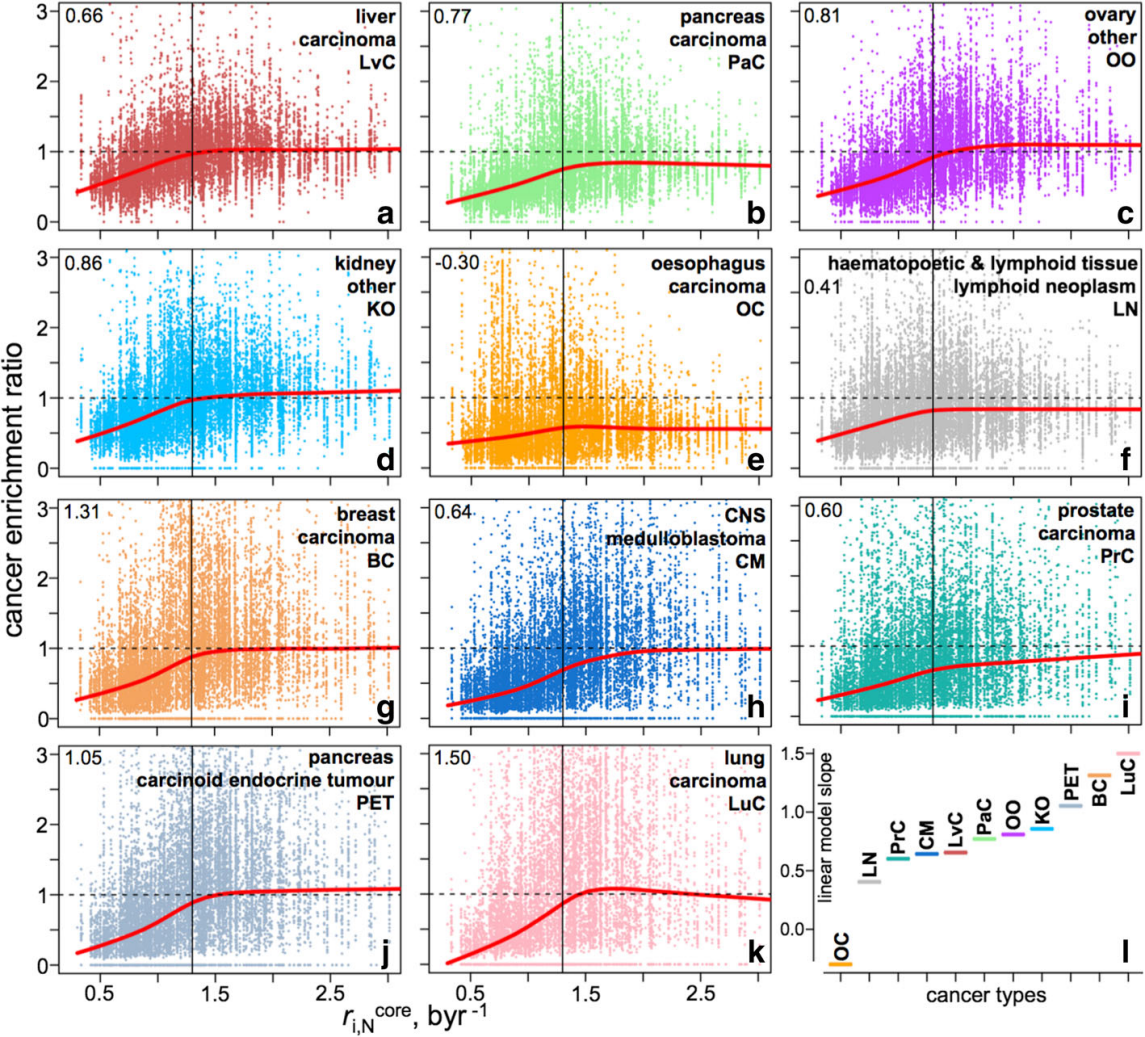
Enrichment of 7-mers with varying BSPs in somatic mutation sites linked to different cancer types. **a**–**k** Each point in the plots corresponds to a unique 7-mer sequence. All the 7-mers that had either C or G of a CpG dyad at the centre were excluded from the plots representing the zoomed [0, 3] byr ^−1^ range of BSPs. The plots (**a**–**k**) show data from 11 cancer types, with their primary tissue and primary cancer types indicated at the top-right corner of each plot. The *red lines* in (**a**–**k**) represent the Lowess [87] smoothing fits, outlining the decrease of the cancer enrichment ratio with the decrease in BSP. The numbers at the top-left corners of the plots show the slopes of the linear fits (not shown) for the data points below the 1.3 byr ^−1^ (*vertical line*), where the depletion of stable k-mers is the most pronounced. The linear model slopes coming from all 11 cancer types are shown in (**l**) for comparing the extent of the cancer enrichment ratio vs. BSP dependence across the analysed cancer types

A recent study, which correlated the cancer and cell division frequencies, suggested that cancers, with their multi-etiologic nature, are linked to random mutation events upon cell division/DNA replication [56], in addition to expressing unique type-dependent mutational signatures [57]. To this end, genomic sites with higher intrinsic BSP (lower stability) may potentially exhibit a higher prevalence of cancer-related genome alterations, as compared to sites of lower intrinsic BSP (higher stability), should the germline and cancer-linked somatic mutations share common mechanisms [54]. Although the sequence context signatures of cancer mutations and their variation across different cancer types is out of the scope of the present work and is covered in detail elsewhere [57–62], here we examined the simple relationship between our calculated germline BSP values and the observed cancer-associated somatic mutations accessed *via* the annotated COSMIC database of somatic mutations in cancer [63] (Methods). Since the Trek data are for the core neutral substitutions, we restricted the analysis to the non-coding and non-polymorphic (not identified as SNP) point mutations (6 mln) in cancer. By mapping these sites to the human genome and retrieving the sequence-context information (7-nt long sequences centred at the mutation points), we processed the data with Trek mapper and obtained the BSP ($r_{i,N}^{core}$) profile for the non-coding sites detected in human cancer. The outcome in Figure S11 in Additional file 1, overlapped with the whole-genome BSP profile, shows that stable sites in the human genome, assigned by the Trek mapper to have $r_{i,N}^{core}$ below 1.13 byr ^−1^, are significantly less likely to undergo somatic mutations in cancer. Like many other disease-causing mutation sites [64], most of the sites that are highly enriched in cancer (Figure S12a in Additional file 1) are CpGs [65], which, even without accounting for the methylation driven increase [12–14] of the mutation rates, show high basal mutability [66]. However, Figure S12b, c in Additional file 1 demonstrates the discussed trend in the 7-mer cancer enrichment ratio (Methods) vs. BSP dependence even when all the CpG sites are removed from the analysis. Furthermore, while investigating the same relationship in different varieties of cancer (as classified based on the primary tissue and primary cancer types, see Methods) we can see that the trend is mostly in place for the 11 cancer types where we have enough data on non-coding somatic mutations (Fig. 8), with the only exception being the oesophageal carcinoma (Fig. 8 e, l). The latter deviation might stem from the greater role of carcinogen driven mechanisms of somatic mutations in a tissue (oesophageal) more exposed to external carcinogens.

Overall, the results show that the intrinsic BSP of different sites in DNA may contribute to their absence/presence in pathological genotypes. In particular, we observe that 7-mers with low germline BSPs of the central base are relatively depleted in cancer-linked somatic mutation data (Figure S12 in Additional file 1 and Fig. 8). They present a cancer enrichment ratio that is smaller than 1, whereas for the unstable 7-mers, the enrichment ratio, on average, tends to 1, meaning that their presence in cancer is overall comparable to the one in the whole genome. Focusing on the average trend (red lines in Fig. 8) that does not expand to the k-mers with > 1 cancer enrichment ratio, we cannot comment about the behaviour of the sites of higher mutability in cancer by the present work. Those could potentially be better reflected in the cancer mutational signature analyses done by others [57, 60, 61]. Our results, however, further outline the potential role of the general imbalance in repair machinery in determining the accumulation of somatic mutations in cancer, where the mutations are originally caused by errors in replication that possibly emerge *via* mechanisms more or less common in somatic and germline cells [54].

### The relation between the Trek $r_{i,j}^{core}$ and germline mutation rates

Since the rates of point mutations can be indirectly estimated by determining the rates at which neutral substitutions accumulate in a genome [5, 51, 67], (also see Box 1 in [68]), and, we have applied stringent criteria for filtering out L1 positions with a potential to be non-neutral, our Trek $r_{i,j}^{core}$ substitution rate constants may serve as contributory estimates for germline mutation rates (especially for the relative mutation rates across individual base conversions). The Trek method complements previous studies on the germline mutation rates [5], and retrieves rate constants (per site, in time domain, byr ^−1^) in a single genome manner, where all the detected substitutions in L1 remnants (their neutral positions only) retrieved from a single genome have accumulated while evolving in multiple (39,894) copies within a lineage of a single organism. This may have a potential to minimise fixation biases and further eliminate biases attributed to the change (mutations) in background mutagenic and reparatory machineries, from an individual to individual, when comparing data from multiple genomes. Taking into account the singleton composition of the unmasked human genome (Additional file 2) and the $r_{i,j}^{sb}$ constants for the individual transitions and transversions brought in Fig. 3, as estimated through Trek method, we can provide a new estimate for the overall germline mutation rate for the human genome to be 1.176 mutations per site per billion year for the used reference human genome. This makes 2.35×10^−8^ mutations per site per generation, assuming 20 years of average generation span [2]. Interestingly, the value is slightly lower than the ∼ 2.5×10^−8^ estimate [2, 5, 67, 69, 70] based on phylogenetic-based approaches, which can be attributed to the more aggressive elimination of biases built in the Trek design (see above). However, the estimate is still greater than the more recent 1.2×10^−8^ to 1.45×10^−8^ evaluations from pedigree-based studies [71–74], known to be free from background recombination influence but highly dependent on the paternal age [75]. Less so for the relative values of the rates for individual base-conversions, we expect the absolute values in Trek to be dependent on the absolute timing of the used five subfamilies of L1 elements. Although the latter reliance may be the cause of the relatedness of the Trek estimate to the phylogeny-based ones, our estimate is still a useful addition to the ongoing debate on calibrating the average germline mutation rates [76–78].

## Conclusions

We have employed a single-genome approach (Trek) that reveals the core ($r_{i,j}^{core}$=$r_{i,j}^{sb} + \delta r_{i,j}^{sr}$) component of the spontaneous single-nucleotide substitution rates and basal substitution propensity constants ($r_{i,N}^{core}$) for the human nuclear genome (Figs. 2 and 3). Although the mobile DNA elements have been used before [20, 40, 46, 79] for estimating averaged substitution rates, the increased quality of the human reference sequence and the detailed subfamily divergence studies for the L1 elements [36–39] done during the past decade enabled the construction of a specific direct method for the single-genome retrieval of the core $r_{i,j}^{core}$ rate constants at a single-nucleotide resolution, while also accounting for the comprehensive short-range context effects beyond the previous estimates for the +1/-1 base effects [35]. The retrieval of our $r_{i,j}^{core}$ data in a single-genome manner adds additional value, since it ensures the absence of potential bias present a) in the comparison of the genomes of different species due to the differences in the molecular machinery (presence of mutations in the respective genes) that influence the overall mutation rates, and b) in the SNP-counting based methods that rely on sites where the visible polymorphism may contain significant selection bias.

Our context-dependent rate constants were then used to drive the equilibration of a random DNA sequence (Fig. 4). The resulting DNA had k-mer (up to 7) contents in better agreement with those of the human genome, as compared to the analogous contents from the calculations based on the singleton substitution rates. The calculated basal substitution propensities revealed motifs that are prone or resistant to substitutions (Fig. 7), and confirmed the presence of a link between core substitution rates in the germline and the somatic mutations in cancer, outlining possible commonalities in the mechanisms of mutations at both levels (Fig. 8). The discussed parallel between the Trek $r_{i,j}^{core}$ substitution rates and the germline mutations makes the observed link with the somatic mutations, inferred from a cancer database, be more logical.

**Fig. 9:**
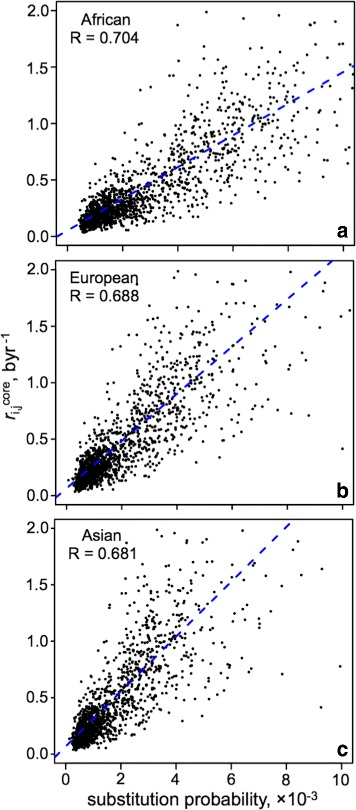
Agreement between the 7-mer context-dependent Trek rate constants (in byr ^−1^), determined in a single-genome manner, and the context-dependent substitution probabilities from the 1000 Genomes Project. **a**–**c** The correlation plots are shown for the substitution probabilities inferred [80] from three different (African, European and Asian) populations [81]. Only 7-mers with data present in both works were used for the comparisons. Overall, the plots highlight the good agreement between the two methods, and show the variation in substitution probabilities across different populations. This variation may be caused by genetic differences in those populations affecting genes/proteins involved in the background mutagenic and repair machineries, thus altering the spontaneous mutation rates

We have become aware of a recently published parallel study [80], where the authors revealed the context-dependent mutation probabilities by analysing the variation reflected in the multiple genomes of the 1000 Genomes Project [81, 82]. By arriving to the CAAT mutable motif (Fig. 7 a) and outlining the importance of the heptameric context (Figure S5 in Additional file 1) in explaining the mutational patterns, the work provided means to additionally validate part of our results by completely different means. Appealingly, the Trek method retrieves rate constants (in time domain, byr ^−1^) in a single genome manner (see above). Besides the potential minimisation of fixation biases, this may provide an opportunity to detect some of the biases attributed to the change (because of mutations) in background mutagenic and repair machineries, from individual to individual, when comparing two or more genomes. In particular, Fig. 9 demonstrates that neutral substitution probabilities may be elevated in one population as compared to the other. The difference was detectable owing to the multi-genomic analyses within each population done in [80]. Trek may thus serve as a single-genome independent and complementary method to assess inter-individual and inter-population variation of the substitution rates, and retrieve the context-dependent rates for species with only single sequenced genome available.

The extended set of core substitution rate constants and the associated program we report may enrich our resources and potentially help advance our understanding of genome dynamics, with possible implications for the role of random substitutions in the emergence of pathological genotypes and the neutral evolution of proteomes.

## Methods

The human reference genome sequence was taken from the Ensembl database (www.ensembl.org), and was of the version hg19/GRCh37. The positions and span of the retrotransposons were taken from the output of the RepeatMasker [83] processing, accessed through the UCSC genome database (www.genome.ucsc.edu). The repeat annotations were those corresponding to the version of the used human RefSeq genome. The R [84] programming language (www.r-project.org) was used for all the consecutive analyses. Most of the computations were performed on the available Linux workstation and computing cluster facilities hosted at the Department of Chemistry, University of Cambridge, and the Cancer Research UK Cambridge Institute. The specific details on the employed methods are brought below, with the general flow and the reasoning behind the approach presented in Results and discussion.

### Revealing the core substitution rate constants

All the remnant sequences of the selected L1 subfamilies (Fig. 1, Table S1 in Additional file 1) were first aligned onto the 6064 nucleotide (nt) reference sequence. As the reference, we took the consensus sequence of the human L1Hs retrotransposon (Fig. 2 a, b, Figures S1 and S2 in Additional file 1). The alignment was done in a pairwise manner with high end-gap penalties (“overlap” mode) that, while allowing insertions and deletions, did not severely break the queried sequences for false mappings with a better global alignment score. R [84] with the Biostrings library for alignment was used. After the alignment, all the relevant substitution fractions were collected for each position in the five L1 subfamilies reporting on a specific time epoch (Fig. 2 b, c). For example, if the position *i* in the reference sequence was G (*b*_1_), the substitution rate constants were calculated for the G →A (*b*_1_→*b*_2_) transition and G →C (*b*_1_→*b*_3_), G →T (*b*_1_→*b*_4_) transversions. First, the base fractions were calculated for five time-reporting L1 subfamilies; i.e. to get the fraction of substitutions accumulated in ∼20.4 myr (age of L1PA5 [37]), all bases in L1PA5 remnants that were precisely mapped on the *i* ^*th*^ position of the reference sequence were counted, and the fractions of G, A, C and T bases retrieved (Fig. 2 c). Here, we applied one of the robustness checks (1^*st*^ stringency criterion) and made sure that the fractions were estimated if at least 700 mapped bases were present for the *i* ^*th*^ position in each time-reporting subfamily (Fig. 2 b, Figure S1 in Additional file 1).

The acceptability of taking 700 as the minimum number of L1 remnants mapped onto a given *i* ^*th*^ position of the L1 reference sequence, while retrieving substitution rate constants, was checked *via* the following test. We divided the L1 sequence pool into two and retrieved the substitution rates twice, in each case using the L1 remnants from the halved pool only. This resulted in two sets of position-specific rate constants, however, with the dataset division resulting in a less number of positions that passed the 700 threshold and the other filtering criteria described below. The high correlation between the two sets (Pearson’s R > 0.9), demonstrated the validity of 700 as a threshold. The high correlation held when decreasing the threshold to 500, decaying after. We have, however, adhered to 700 to ensure the quality of the final values.

We also aimed at calculating such substitution rates for only the positions where the substitutions are neutral and not specifically selected for or against (2^*nd*^ stringency criterion). In other words, the position should not be a polymorphic or a subfamily speciation-defining nucleotide. We filtered out such cases by ensuring that any eligible *i* ^*th*^ position had the same nucleotide of the reference sequence as its most prevalent variant with a minimum of 80% occurrence in all subfamilies (Fig. 2 c, Figure S1 in Additional file 1).

The 80% threshold was taken from the following consideration. The average crude single-nucleotide substitution rate is noted to be 12.85×10^−9^ substitutions per site per generation [4]. Assuming an average generation length of 20 years [2], the substitution rate constant in a time domain can be crudely approximated as 0.64 byr ^−1^. In the course of 20.4 myr (the age of L1PA5), this should result in only a 1.31% substituted base fraction at a given site, caused by the average spontaneous substitution rates. Therefore, by assuming a threshold of 80%, we allow up to 15 times the variation of the rates from the average estimate, which is a safe range [4] for the direct estimation of the single-nucleotide substitution rates and their core variation.

Having the substitution fraction data, from five different ages and for three (*b*_1_→*b*_2_, *b*_1_→*b*_3_, *b*_1_→*b*_4_) possible substitutions at the position *i*, allowed the fitting of a linear model *via* the least squares methodology for the fraction-versus-time dependence for each substitution separately (Fig. 2 d). If the data, hence the fitted line, were of high quality, the slope was expected to represent the $r_{i,j}^{core}$ substitution rate constant. We applied the final robustness filtering at this stage, by making sure that the rates were calculated for only the cases where the time correlation of the substitution fractions in Fig. 2 d had greater than 0.7 Pearson’s correlation coefficient (3^*rd*^ stringency criterion, Figure S1 in Additional file 1). This ensured that the retrieved fractions of the substitutions comprised of only the time-accumulated substitutions, rather than of targeted substitutions during the active life-span of the L1 elements, before their silencing.

Please note, however, that the correlation coefficients in most of such time correlations that passed the whole Trek procedure were substantially higher (the observed Pearson’s correlation coefficients were centred at 0.92 with 0.07 standard deviation). Furthermore, the resulting slope (rate constant) estimates had significantly high *t*-values, averaged at 6.2, showing that the standard error in estimates was, on average, 6.2 times smaller than the estimated value. The individual *t*-values are presented in the Additional file 2 along with the rate constants.

The procedure was done for all the 6064 positions in the L1 reference sequence, except the positions 5856–5895 and 6018–6064, close to the 3’-end (Figure S2 in Additional file 1) that engulf low-complexity G-rich and A-rich sequences correspondingly, prone to alignment errors.

One of the reasons for the usage of only the young L1 subfamilies (spanning 20.4 myr age) was to minimise the potential error in rate constant determination in the Trek procedure caused by repeated substitutions hitting the same position during the considered period of the substitution accumulation. The effect is indeed negligible for 20.4 myr span, as we can estimate using the above mentioned 0.64 byr ^−1^ value [4] for the average *i*→*j* substitution rate constant, *r*. Since the rate constant is sufficiently small to induce only a small *δ* *f*_*j*_ change in substituted base fraction during the *δ* *t* = 0.0204 byr (20.4 myr) time period (see above), we can equate the *δ* *f*_*j*_ change in the fraction of the base *j* (at the given position that had the original base *i* identity in a large population of homologous sequences) to the *p*≈*δ* *f*_*j*_≈*r* *δ* *t* substitution probability within *δ* *t* period. We can thus make a crude estimation for the probability of the second substitution to another, *k*≠*j*, base happening at the same position to be (*r* *δ* *t*)^2^, which is the product of individual substitution probabilities assuming that the rate constant does not change from our average estimate *r* across those two substitution types. We can permit this for the sake of the back-of-the-envelope estimation of the order of the effect expected from the repeated substitutions hitting the same site within 20.4 myr period. To this end, the $\delta f_{j}^{app}$ apparent change in *i*→*j* substitution fraction that we would observe by neglecting the additional *j*→*k* substitution, would underestimate the more realistic *δ* *f*_*j*_ and be equal to $\delta f_{j}^{app}$= *r* *δ* *t*- (*r* *δ* *t*)^2^, as we would not count the *j* bases that emerged but became additionally substituted by *k*. Hence, the corresponding apparent rate constant that neglects second substitution would also underestimate the actual value *r*, and can be expressed as *r* ^*app*^=$\delta f_{j}^{app}/\delta t$= (*r* *δ* *t*- (*r* *δ* *t*)^2^)/*δ* *t*= *r*(1- *r* *δ* *t*). This means that the underestimation of the actual rate constant would be by [*r*- *r*(1- *r* *δ* *t*)]×100/*r*= 100×*r* *δ* *t* %. Putting 0.64 byr ^−1^ for *r* and the 0.0204 byr for *δ* *t*, we can expect only 1.3% contribution to rate constants from the repeated second substitution at the same position within 20.4 myr. Since some of the other non-*j* bases could revert to *j*s and balance the underestimation of the *δ* *f*_*j*_ fraction, the error could be even smaller. This shows that repeated substitutions could be neglected in our 20.4 myr time-scale. Furthermore, the validity of the $r_{i,j}^{core}$ rate constants (Fig. 3) was further checked through the independent analyses reflected in Figure S3 in Additional file 1 and Fig. 9.

### Finding the influence range of neighbour nucleotides

We have used gradient boosting machines (GBM) [48, 49] to elucidate the effective range for the core sequence-context effects. This was achieved by developing test models to evaluate the predictive strength of only the neighbouring bases in defining the core substitution rate of the central base. Tree-based gradient boosting machines (GBM) are a class of machine learning methodologies that produce strong regression or classification models by creating an ensemble of weaker models. The technique consecutively adds weaker models in the forms of decision trees that aim to reduce the residuals of the predictions [48, 49]. It was used as implemented in the gbm library for R. For each *i*→*j* substitution type, all the found Trek data were taken without the possible outliers, which were filtered by allowing only the usage of the values that were within the 1.65 × standard deviation range (keeps ∼90% data if normally distributed) of the constants in a given substitution category. The sequences were then processed to produce *p* *o* *s*/*b*_*i*_ decoupled features that were associated with the relative adjacent positions (*pos*, - for upstream and + for downstream positions) and their possible four *b*_*i*_ base types. Those features took values 0 or 1, depending on whether the base at an associated relative position was of the *b*_*i*_ (1) or any other base type (0). For instance, if we want to develop a model accounting for only a single upstream (*pos* = −1) and a single downstream (*pos* = +1) nucleotides, hence predicting the substitution rates for different 3-mers, where the central base is the one that mutates, then we would produce 8 *p* *o* *s*/*b*_*i*_ features for the GBM fitting. There, 4 binary features (-1/A, -1/C, -1/G and -1/T) would describe whether the upstream -1 position is of base type A, C, G or T, and 4 binary features (*p* *o* *s*/*b*_*i*_) would describe the same for the downstream +1 position. We built the models using 3-, 5-, 7-, 9-, and 11-mers, thus accounting for one, two, three, four and five upstream and the same number of downstream neighbour bases (Figure S4 in Additional file 1). The absence of the coupling in the binary features, unlike in the case where, for instance, one employs only two binary features per 4 states, enabled us to also investigate the predictive significance of each nucleobase identity at a given neighbouring position, which was useful in deciding against the construction of more complex machine learning models (see below) using additional features with higher level of abstraction for the sequence information (overall base content, sequence-derivative properties). The GBM models were then fitted by systematically trying different permutations of the tuning values [49] for the number of trees (50-7500), interaction depth (1-10), shrinkage (0.001, 0.01 and 0.1), the number of minimum observations per node (1-28) and the bag fraction (0.25-0.65). The optimal combinations of the tuning parameters were found per substitution type and sequence length, *via* a 16-fold cross validation repeated 7 times. The found best parameters are accessible in the Additional file 2, and the predictive performances of the best models, from the repeated cross validation studies, are presented in Figure S5 in Additional file 1. To make a predictor of $r_{i,j}^{core}$ based on a sequence only, we, however, found it much better to use direct averaged values coming from the proposed Trek methodology for each k-mer, rather than the GBM models, as the Trek values are already well averaged across multiple occurrences of the same sequence in different loci of the human genome (Figs. 1, 2 and 3, Figure S3 in Additional file 1). Furthermore, the overall poor performance of the decision-tree-based GBM models implies that the influence of the immediate context is highly non-additive and non-Bayesian, which is expected taking into account the nature of the core context-dependent mutations. The latter reflect the intrinsic short-range sequence properties, interactions and recognition with the overall mutagenic and repair machinery present in a given organism. There, the whole sequence at a certain small (< 10 nt) scale [9] is what likely defines the interaction [11], and it is hard to represent such effects through even smaller-scale constituents linked with each other at a certain dimension. The direct model-free approach used in our Trek mapper methodology (see below) thus seems preferable in mapping the $r_{i,j}^{core}$ rate constants throughout the human genome. To this end, the GBM models here had a sole purpose of identifying the optimal range of influence for accounting the neighbouring nucleotides. The optimal range was found to be captured, on average, by a 5-7-nt long window (Figures S4 and S5 in Additional file 1) which is in an excellent agreement with the prior < 10 nt estimate [9–11, 44, 50]. We thus used the maximum 7-nt length to stratify the Trek data for the further model-free mapping on any provided sequence, including the whole human genome.

### Mapping the Trek substitution data

We developed an open source Trek mapper program. For each *i* position in a query sequence (Figure S6 in Additional file 1), the program looks at the bases *i*−3 to *i*+3. If the exact 7-mer, with the associated rate constant values, is not available in the Trek database, the program reduces the size of the sequence to 5, by considering *i*−2 to *i*+2 positions, or, if necessary, to 3- or 1-mers, until an exact match is found in the database. This would mean that some reported substitution rates would come from the actual triad data. In any case, about half will come from pentads and some from heptads, accounting for more precise sequence-context information (see Results and discussion for the numbers). A few will originate from the fully averaged single-base (1-mer) Trek rates. For each unique sequence in the discussed 7-, 5-, 3- and 1-mers, if the k-mer appears more than once in the reference L1 sequence, of course with different neighbours at the positions out of the k-mer range, we average the Trek values by taking the median. For instance, the *r*_*G*,*A*_ substitution rate constant in the 3-mer AGT represents the rate averaged across all the appearances of AGT in the L1 reference sequence, which would normally be with varying other neighbour bases, out of the 3-mer range. The *r*_*G*,*A*_ in AGT will therefore represent the average rate constant across all the representatives of the significant range, the NNAGTNN 7-mers, present in L1, where N can be any of the four bases. In the same way, the substitution rate constants for the single bases (1-mers) can be considered as fully averaged across all the possible neighbour effects in NNNGNNN sequences. Our algorithm therefore makes the most of transposon exposed substitution rate data of the human genome, returning the best possible values inferable from our Trek database and, where uncertainty is present, returning the best averaged values for a shorter context range. Furthermore, we have enabled the usage of symmetrized Trek parameterisation, assuming an overall strand-invariance of the substitution rates. In the latter case, the complementary rate constants of the central bases in two reverse complementary k-mers were equalised. For example, the G →A substitution rate constant (*r*_1_) in the 3-mer AGC was set equal to the complementary C →T rate constant (*r*_2_) in the reverse complementary GCT. The data equalisation was done in the following way: if both *r*_1_ and *r*_2_ were of the same quality accounting for the whole sequence-context information in both 3-mer variants, then both values were set to (*r*_1_+*r*_2_)/2; however, if one of the rate constants was determined with a better quality, since the full 3-mer data for the other case was missing and the 1-mer average was used as a replacement, then the rate constant of the better quality variant was assigned to both *r*_1_ and *r*_2_. Accounting for the strand symmetry improves the results of the validation studies, further refining the substitution rate constant values and increasing the coverage of longer k-mers in the Trek database. The described open source program, along with the associated data can be accessed through the *http://trek.atgcdynamics.org* web page. Future improvements in data and the program, through extending the types of mobile DNA in the Trek procedure, will be reflected on the same web site. The Trek mapper server application was written in R, using the Shiny library and server backend (http://shiny.rstudio.com). The stand-alone program, supporting both graphical and programmatic (terminal) interaction and multi-processor computing, can be obtained from the same web page, to be used for larger projects and genomes.

### Equilibration of a random DNA sequence with Trek rates

A 5 million (mln) nt sized random sequence (in silico “genome”) was created with the initial A, T, G and C base contents set to 20, 20, 30 and 30% correspondingly, hence with 60% genomic G+C content. The length was selected to cope with the finite computational and time resources, though operating on lengthier sequences will not change the outcome of the calculations, since the captured sequence-context effects are within 7-nt window. We first calculated the probabilities of all the possible substitution in this random sequence, which basically meant the assignment of three substitution rate constants per position in the genome, describing the conversion into the three bases other than the base already present in the respective position. This was done using the Trek mapper described above. Next, at each step, we sampled 5000 substitution weighted by the calculated 3×5 mln rate constants. We then identified those 5000 positions and the corresponding substitution types that were sampled to happen (Figure S7 in Additional file 1), performed those substitutions, and, updated the probability values *via* the Trek mapper. Repeated multiple times, the process evolved the sequence ruled by the core spontaneous substitution rate constants that are sensitive to the changes in the sequence composition at the immediate vicinity in the genome (Figs. 4, 5 and 6, Figure S8 in Additional file 1). The same simulation was also done by using the substitution rates averaged to singletons only, thus with no context dependence (Fig. 6).

For the comparison of the simulated sequence at equilibrium with the real human genome (RefSeq), we calculated the fractions of different oligomers (k-mers) in both sequences (Additional file 2). The k-mer contents of the human genome were calculated by sliding a window of size k (from 1 to 7) and counting the occurrence of each 4^*k*^ unique sequence. We used a direct calculation of the lexicological index [85] of a string to increase the computational efficiency of the k-mer counting. Although, data from the masked human genome were used in the k-mer analyses to rule out any bias from the presence of the same L1 elements in the object of application of Trek data, the comparison of the masked and unmasked genomes showed only negligible differences in both single base and short k-mer contents. If, however, we consider only the L1 elements, the k-mer content in L1 is markedly different from the rest of the genome (Figure S9 in Additional file 1), additionally signifying the absence of circularity in our procedure and results.

### Basal substitution propensity at cancer-linked sites

The basal substitution propensities were defined and retrieved for the human genome as presented in the Results and discussion (Fig. 7, Figure S10 in Additional file 1). We took all the non-coding somatic point mutation data associated with cancer from the COSMIC database [63] (http://cancer.sanger.ac.uk/cosmic, NCV dataset accessed in February, 2015). Since our Trek rate constants are for the spontaneous core substitutions, we only considered the sites that were also not declared as known SNPs (the status was present in the NCV dataset). This was to ensure that we excluded sites where an active polymorphism is potentially encouraged by natural selection. About 3.7% data from the remaining set of cancer-linked somatic mutations were duplicates, with no differences found in genomic location and mutation types. We removed those, keeping only the single first-encountered copies of such entries (Figure S11 in Additional file 1). The resulting data contained 5,984,711 mutation entries.

The cancer enrichment score (Figure S12 in Additional file 1) for a given k-mer sequence was calculated by taking the ratio of the occurrence fractions, calculated in (numerator) only the cancer-linked sites (where the linked base is the central one in the k-mer) and (denominator) in the whole repeat-masked human genome (Additional file 2). We next repeated the above analysis by examining non-coding somatic mutations from different types of cancer (Fig. 8), as guided by the primary tissue (primT) and primary cancer (primC) types recorded in the COSMIC database. To stratify data, we examined the primT:primC pair as a cancer type identifier for each mutation. The data contained 29 unique primT:primC pairs, of which 11 had a substantial number of records (2,422,060 non-coding mutations for liver:carcinoma, 1,287,384 for pancreas:carcinoma, 851,028 for ovary:other, 411,076 for kidney:other, 331,520 for oesophagus:carcinoma, 286,800 for haematopoetic and lymphoid tissue:lymphoid neoplasm, 92,276 for breast:carcinoma, 79,342 for central nervous system (CNS):primitive neuroectodermal tumour/medulloblastoma, 69,721 for prostate:carcinoma, 62,714 for pancreas:carcinoid endocrine/tumour, 38,835 for lung carcinoma). This list was followed by 19,004 records with non-specified primary tissue and cancer types, and substantially low number of records for the rest (at or below ∼10 k records). We thus analysed the results from the top 11 identifiers with the most number of recorded somatic mutations.

## Additional files

Additional file 1
**Supplementary note, table and figures.** Additional supporting note, table and figures referenced in the text (**Note S1**, **Table S1**, **Figures S1**-**S12**), as well as the detailed description of the Additional file 2 content. The file is in the PDF format. (PDF 3307 kb)

Additional file 2
**Supplementary numerical data.** The raw data on the substitution rate constants in the reference L1 sequence (data 1), the resulting Trek database processed with (data 2) and without (data 3) the strand-symmetry considerations, the k-mer content for the masked and unmasked human genomes (data 4), the full set of 7-mer sequences with the respective cancer enrichment scores and basal substitution propensity values (data 5), and the GBM parameters that were minimising the error of the tree-based test models (data 6). The file is in the plain TXT format. (TXT 3266 kb)

## Abbreviations

bln: Billion
BSP: Basal substitution propensity
byr: Billion years
COSMIC: Catalogue of somatic mutations in cancer
GBM: Gradient boosting machines
L1: Long interspersed nuclear element 1
LINE: Long interspersed nuclear element
LTR: Long terminal repeat
mln: Million
myr: Million years
nt: Nucleotide
SINE: Short interspersed nuclear element
SNP: Single-nucleotide polymorphism

## References

[1] Lynch M (2007). The Origins of Genome Architecture.

[2] Nachman MW, Crowell SL (2000). Estimate of the mutation rate per nucleotide in humans. Genetics.

[3] Chen JQ, Wu Y, Yang H, Bergelson J, Kreitman M, Tian D (2009). Variation in the ratio of nucleotide substitution and indel rates across genomes in mammals and bacteria. Mol Biol Evol.

[4] Lynch M (2010). Rate, molecular spectrum, and consequences of human mutation. Proc Natl Acad Sci USA.

[5] Ségurel L, Wyman MJ, Przeworski M (2014). Determinants of mutation rate variation in the human germline. Annu Rev Genom Hum Genet.

[6] Denver DR, Morris K, Lynch M, Vassilieva L, Thomas K (2000). High direct estimate of the mutation rate in the mitochondrial genome of caenorhabditis elegans. Science.

[7] Lynch M, Sung W, Morris K, Coffey N, Landry CR, Dopman EB, Dickinson WJ, Okamoto K, Kulkarni S, Hartl DL, Thomas WK (2008). A genome-wide view of the spectrum of spontaneous mutations in yeast. Proc Natl Acad Sci USA.

[8] Zhu YO, Siegal ML, Hall DW, Petrov DA (2014). Precise estimates of mutation rate and spectrum in yeast. Proc Natl Acad Sci USA.

[9] Silva JC, Kondrashov AS (2002). Patterns in spontaneous mutation revealed by human-baboon sequence comparison. Trends Genet.

[10] Ellegren H, Smith NG, Webster MT (2003). Mutation rate variation in the mammalian genome. Curr Opin Genet Devel.

[11] Zavolan M, Kepler TB (2001). Statistical inference of sequence-dependent mutation rates. Curr Opin Genet Devel.

[12] Sved J, Bird A (1990). The expected equilibrium of the CpG dinucleotide in vertebrate genomes under a mutation model. Proc Natl Acad Sci USA.

[13] Jiang C, Zhao Z (2006). Directionality of point mutation and 5-methylcytosine deamination rates in the chimpanzee genome. BMC Genomics.

[14] Supek F, Lehner B, Hajkova P, Warnecke T (2014). Hydroxymethylated cytosines are associated with elevated C to G transversion rates. PLoS Genet.

[15] Majewski J, Ott J (2002). Distribution and characterization of regulatory elements in the human genome. Genome Res.

[16] Hellmann I, Zollner S, Enard W, Ebersberger I, Nickel B, Paabo S (2003). Selection on human genes as revealed by comparisons to chimpanzee cDNA. Genome Res.

[17] Fryxell KJ, Moon WJ (2005). CpG mutation rates in the human genome are highly dependent on local GC content. Mol Biol Evol.

[18] Mugal CF, Ellegren H (2011). Substitution rate variation at human CpG sites correlates with non-CpG divergence, methylation level and GC content. Genome Biol.

[19] Lercher MJ, Hurst LD (2002). Human SNP variability and mutation rate are higher in regions of high recombination. Trends Genet.

[20] Arndt PF, Hwa T, Petrov DA (2005). Substantial regional variation in substitution rates in the human genome: importance of GC content, gene density, and telomere-specific effects. J Mol Evol.

[21] Duret L, Arndt PF (2008). The impact of recombination on nucleotide substitutions in the human genome. PLoS Genet.

[22] Hanawalt PC, Spivak G (2008). Transcription-coupled DNA repair: two decades of progress and surprises. Nat Rev Mol Cell Biol.

[23] Gaillard H, Herrera-Moyano E, Aguilera A (2013). Transcription-associated genome instability. Chem Rev.

[24] Schuster-Böckler B, Lehner B (2012). Chromatin organization is a major influence on regional mutation rates in human cancer cells. Nature.

[25] Agier N, Fischer G (2012). The mutational profile of the yeast genome is shaped by replication. Mol Biol Evol.

[26] Reijns MAM, Kemp H, Ding J, de Procé SM, Jackson AP, Taylor MS (2015). Lagging-strand replication shapes the mutational landscape of the genome. Nature.

[27] Supek F, Lehner B (2015). Differential DNA mismatch repair underlies mutation rate variation across the human genome. Nature.

[28] Ellison CE, Bachtrog D (2015). Non-allelic gene conversion enables rapid evolutionary change at multiple regulatory sites encoded by transposable elements. Elife.

[29] Ellegren H (2007). Characteristics, causes and evolutionary consequences of male-biased mutation. Proc Roy Soc. B.

[30] Subramanian S, Kumar S (2003). Neutral substitutions occur at a faster rate in exons than in noncoding DNA in primate genomes. Genome Res.

[31] Chamary JV, Parmley JL, Hurst LD (2006). Hearing silence: non-neutral evolution at synonymous sites in mammals. Nat Rev Genet.

[32] McVean GT, Hurst LD (1997). Evidence for a selectively favourable reduction in the mutation rate of the X chromosome. Nature.

[33] Martincorena I, Luscombe NM (2012). Non-random mutation: the evolution of targeted hypermutation and hypomutation. BioEssays.

[34] Kazazian Jr HH (2011). Mobile DNA. Finding Treasure in Junk.

[35] Hwang DG, Green P (2004). Bayesian Markov chain Monte Carlo sequence analysis reveals varying neutral substitution patterns in mammalian evolution. Proc Natl Acad Sci USA.

[36] Boissinot S, Chevret P, Furano AV (2000). L1 (LINE-1) retrotransposon evolution and amplification in recent human history. Mol Biol Evol.

[37] Khan H (2006). Molecular evolution and tempo of amplification of human LINE-1 retrotransposons since the origin of primates. Genome Res.

[38] Lee J, Cordaux R, Han K, Wang J, Hedges DJ, Liang P, Batzer MA (2007). Different evolutionary fates of recently integrated human and chimpanzee LINE-1 retrotransposons. Gene.

[39] Giordano J, Ge Y, Gelfand Y, Abrusán G, Benson G, Warburton PE (2007). Evolutionary history of mammalian transposons determined by genome-wide defragmentation. PLoS Comput. Biol.

[40] Lander et al.Initial sequencing and analysis of the human genome. Nature. 2001; 409(6822):860–921. 10.1038/35057062 11237011

[41] Medstrand P, van de Lagemaat LN, Mager DL (2002). Retroelement distributions in the human genome: variations associated with age and proximity to genes. Genome Res.

[42] Rawal K, Ramaswamy R (2011). Genome-wide analysis of mobile genetic element insertion sites. Nucl Acids Res.

[43] Duret L, Marais G, Biémont C (2000). Transposons but not retrotransposons are located preferentially in regions of high recombination rate in Caenorhabditis elegans. Genetics.

[44] Nevarez PA, DeBoever CM, Freeland BJ, Quitt MA, Bush EC (2010). Context dependent substitution biases vary within the human genome. BMC Bioinform.

[45] Criscione SW, Zhang Y, Thompson W, Sedivy JM, Neretti N (2014). Transcriptional landscape of repetitive elements in normal and cancer human cells. BMC Genomics.

[46] Arndt PF, Petrov DA, Hwa T (2003). Distinct changes of genomic biases in nucleotide substitution at the time of Mammalian radiation. Mol. Biol. Evol.

[47] Lemey P, Salemi M, Vandamme AM, editors. (2012). The Phylogenetic Handbook: a Practical Approach to the Phylogenetic Analysis and Hypothesis Testing.

[48] Friedman JH. Greedy function approximation: a gradient boosting machine. Reitz Lecture, IMS. 1999:1–39. http://statweb.stanford.edu/~jhf/ftp/trebst.pdf.

[49] Kuhn M, Johnson K (2013). Applied Predictive Modeling.

[50] Zhao Z, Boerwinkle E (2002). Neighboring-nucleotide effects on single nucleotide polymorphisms: A study of 2.6 million polymorphisms across the human genome. Genome Res.

[51] Kimura M (1983). The Neutral Theory of Molecular Evolution.

[52] Vitti JJ, Grossman SR, Sabeti PC (2013). Detecting natural selection in genomic data. Annu. Rev. Genet.

[53] Sung W, Ackerman MS, Gout JF, Miller SF, Williams E, Foster PL, Lynch M (2015). Asymmetric context-dependent mutation patterns revealed through mutation-accumulation experiments. Mol Biol Evol.

[54] Ju et al.Origins and functional consequences of somatic mitochondrial DNA mutations in human cancer. Elife. 2014; 3:02935. 10.7554/eLife.02935 PMC4371858 25271376

[55] Schneider TD, Stephens RM (1990). Sequence logos: a new way to display consensus sequences. Nucl Acids Res.

[56] Tomasetti C, Vogelstein B (2015). Cancer etiology. Variation in cancer risk among tissues can be explained by the number of stem cell divisions. Science.

[57] Alexandrov et al.Signatures of mutational processes in human cancer. Nature. 2013; 500(7463):415–21. 10.1038/nature12477 PMC3776390 23945592

[58] Hodgkinson A, Chen Y, Eyre-Walker A (2012). The large-scale distribution of somatic mutations in cancer genomes. Human Mutat.

[59] Kandoth C, McLellan MD, Vandin F, Ye K, Niu B, Lu C, Xie M, Zhang Q, McMichael JF, Wyczalkowski MA, Leiserson MDM, Miller CA, Welch JS, Walter MJ, Wendl MC, Ley TJ, Wilson RK, Raphael BJ, Ding L (2013). Mutational landscape and significance across 12 major cancer types. Nature.

[60] Alexandrov LB, Nik-Zainal S, Wedge DC, Campbell PJ, Stratton MR (2013). Deciphering signatures of mutational processes operative in human cancer. Cell Rep.

[61] Fischer A, Illingworth CJR, Campbell PJ, Mustonen V (2013). EMu: probabilistic inference of mutational processes and their localization in the cancer genome. Genome Biol.

[62] Jia P, Pao W, Zhao Z (2014). Patterns and processes of somatic mutations in nine major cancers. BMC Med Genom.

[63] Forbes SA, Bindal N, Bamford S, Cole C, Kok CY, Beare D, Jia M, Shepherd R, Leung K, Menzies A, Teague JW, Campbell PJ, Stratton MR, Futreal PA (2011). COSMIC: mining complete cancer genomes in the catalogue of somatic mutations in cancer. Nucl Acids Res.

[64] Cooper DN, Krawczak M (1990). The mutational spectrum of single base-pair substitutions causing human genetic disease: patterns and predictions. Human Genet.

[65] Greenman et al.Patterns of somatic mutation in human cancer genomes. Nature. 2007; 446(7132):153–8. 10.1038/nature05610 PMC2712719 17344846

[66] Rubin AF, Green P (2009). Mutation patterns in cancer genomes. Proc Natl Acad Sci USA.

[67] Kumar S, Subramanian S (2002). Mutation rates in mammalian genomes. Proc Natl Acad Sci USA.

[68] Barrick JE, Lenski RE (2013). Genome dynamics during experimental evolution. Nat Rev Genet.

[69] Campbell CD, Eichler EE (2013). Properties and rates of germline mutations in humans. Trends Genet.

[70] Shendure J, Akey JM (2015). The origins, determinants, and consequences of human mutations. Science.

[71] Kong A, Frigge ML, Masson G, Besenbacher S, Sulem P, Magnusson G, Gudjonsson SA, Sigurdsson A, Jonasdottir A, Jonasdottir A, Wong WSW, Sigurdsson G, Walters GB, Steinberg S, Helgason H, Thorleifsson G, Gudbjartsson DF, Helgason A, Magnusson OT, Thorsteinsdottir U, Stefansson K (2012). Rate of de novo mutations and the importance of father’s age to disease risk. Nature.

[72] Fu Q, Li H, Moorjani P, Jay F, Slepchenko SM, Bondarev AA, Johnson PLF, Aximu-Petri A, Prüfer K, de Filippo C, Meyer M, Zwyns N, Salazar-García DC, Kuzmin YV, Keates SG, Kosintsev PA, Razhev DI, Richards MP, Peristov NV, Lachmann M, Douka K, Higham TFG, Slatkin M, Hublin JJ, Reich D, Kelso J, Viola TB, Pääbo S (2014). Genome sequence of a 45,000-year-old modern human from western Siberia. Nature.

[73] Rahbari R, Wuster A, Lindsay SJ, Hardwick RJ, Alexandrov LB, Al Turki S, Dominiczak A, Morris A, Porteous D, Smith B, Stratton MR, Hurles ME, UK10K Consortium (2016). Timing, rates and spectra of human germline mutation. Nat Genet.

[74] Narasimhan VM, Rahbari R, Scally A, Wuster A, Mason D, Xue Y, Wright J, Trembath RC, Maher ER, van Heel DA, Auton A, Hurles ME, Tyler-Smith C, Durbin R. A direct multi-generational estimate of the human mutation rate from autozygous segments seen in thousands of parentally related individuals. 2016. BioRxiv http://dx.doi.org/10.1101/059436.

[75] Francioli LC, Polak PP, Koren A, Menelaou A, Chun S, Renkens I, van Duijn CM, Swertz M, Wijmenga C, van Ommen G, Slagboom PE, Boomsma DI, Ye K, Guryev V, Arndt PF, Kloosterman WP, de Bakker PIW, Sunyaev SR, Genome of the Netherlands Consortium (2015). Genome-wide patterns and properties of de novo mutations in humans. Nat Genet.

[76] Lipson M, Loh PR, Sankararaman S, Patterson N, Berger B, Reich D (2015). Calibrating the human mutation rate via ancestral recombination density in diploid genomes. PLoS Genet.

[77] Callaway E (2015). DNA clock proves tough to set. Nature.

[78] Moorjani P, Gao Z, Przeworski M. Human germline mutation and the erratic molecular clock. 2016. BioRxiv http://dx.doi.org/10.1101/058024. 10.1371/journal.pbio.2000744 PMC5070741 27760127

[79] Walser JC, Ponger L, Furano AV (2008). CpG dinucleotides and the mutation rate of non-CpG DNA. Genome Res.

[80] Aggarwala V, Voight BF (2016). An expanded sequence context model broadly explains variability in polymorphism levels across the human genome. Nat Genet.

[81] 1000 Genomes Project Consortium: (2010). A map of human genome variation from population-scale sequencing. Nature.

[82] 1000 Genomes Project Consortium etal.A global reference for human genetic variation. Nature. 2015; 526(7571):68–74. 10.1038/nature15393 PMC4750478 26432245

[83] Smit AFA, Hubley R, Green P. RepeatMasker Open-4.0. 2015. http://www.repeatmasker.org.

[84] R Core Team:. R: a language and environment for statistical computing. 2015.

[85] Compeau P, Pevzner P (2014). Bioinformatics Algorithms: an Active Learning Approach.

[86] Sahakyan AB, Balasubramanian S. Core variability in substitution rates and the basal sequence characteristics of the human genome. 2015. BioRxiv http://dx.doi.org/10.1101/024257.

[87] Cleveland WS (1979). Robust locally weighted regression and smoothing scatterplots. J Am Stat Assoc.

