# Supplementary material for: Single genome retrieval of context-dependent variability in mutation rates for human germline
Source: BMC Genomics. 2017 Jan 13;18:81. doi: 10.1186/s12864-016-3440-5 (PMC5237266; doi:10.1186/s12864-016-3440-5)
Supplement: Additional file 1 — Supplementary note, table and figures. Additional supporting note, table and figures referenced in the text (Note S1, Table S1, Figures S1-S12), as well as the detailed description of the Additional file 2 content. The file is in the PDF format. (PDF 3307 kb) [file 12864_2016_3440_MOESM1_ESM.pdf]

Single genome retrieval of context-dependent variability in mutation rates for human germline

Aleksandr B. Sahakyan[1,*] and Shankar Balasubramanian[1,2,3,*]

[1]Department of Chemistry, University of Cambridge, Lensfield Road, Cambridge CB2 1EW, UK.
[2]Cancer Research UK Cambridge Institute, University of Cambridge, Li Ka Shing Centre, Robinson Way, Cambridge CB2 0RE, UK.
[3]School of Clinical Medicine, University of Cambridge, Cambridge CB2 0SP, UK.

*Correspondence to as952@cam.ac.uk (A.B.S.) and sb10031@cam.ac.uk (S.B.)

Additional file 1: Supplementary Note, Table and Figures

Content

Note S1	---	2
Table S1	---	4
Figure S1	---	5
Figure S2	---	6
Figure S3	---	7
Figure S4	---	8
Figure S5	---	9
Figure S6	---	10
Figure S7	---	11
Figure S8	---	12
Figure S9	---	13
Figure S10	---	14
Figure S11	---	15
Figure S12	---	16
Description of the Additional file 2	---	17
Supplementary references	---	18

Note S1. Installation and usage of Trek

To install the Trek program, the following steps should be taken:

1. Install the latest version of R programming language or skip to the next step.

2. Launch R from the command line and install the R packages *shiny* (required for the graphical user interface), *doMC*, *foreach* and *itertools* (required for a parallel execution of the program) from within R.

```
$ R
```

```
> install.packages("shiny")
> install.packages("doMC")
> install.packages("foreach")
> install.packages("itertools")
```

3. Download the Trek source code from the GitHub repository. This can also be done via a Linux/Unix/OSX command line (given that git is installed) by typing the following:

```
$ git clone https://github.com/aleksahak/Trek
```

The downloaded folder has the following content:

- **lib/** - subfolder containing all the source files,
- **TrekGUI/** - subfolder containing the graphical user interface,
- **Trek.R** - interfacing R script used to execute Trek from command line.

4. Finally, the package should be bit compiled by going into the **lib/** subfolder and executing **bitcompile.R** code from within R.

```
$ cd lib/
$ R
```

```
> source("bitcompile.R")
```

This generates a single file, **Trek.lib**, which encapsulates the main Trek code and all its dependencies. At this stage, the subfolder **lib/** can be safely removed. The user might, however, want to copy the **test.fasta** file from inside **lib/**, in order to test the Trek installation. At this point, the Trek installation folder should contain:

- **Trek.lib** - the bit-compiled Trek program,
- **TrekGUI/** - subfolder containing the graphical user interface,
- **Trek.R** - the interfacing R script used to execute Trek from command line,

and, if the test sequence file is preserved,

- **test.fasta** - the example DNA sequence fasta file.

Trek can be executed as an R program, either from within R, or from the Linux/Unix/OSX command

line through R CMD BATCH or Rscript execution. The latter two options allow the usage of Trek from the scripts written via programming languages other than R.

In R, as exemplified in the **Trek.R** interfacing script, one should load the **Trek.lib** bit-compiled file, then execute Trek via the R function *Trek()*. The latter accepts four arguments:

- *FastaFile* - the relative or absolute path to the fasta file to analyse,
- *OutFile* - the relative or absolute path to the output file to be saved,
- *MutRates* - an argument accepting “sym” and “nosym” options for the strand-symmetrised (recommended) and raw parameter usage for substitution rates,
- *nCPU* - the number of CPUs to be used for the calculation, where the larger values can markedly speed up the mapping process for entire genomes.

Alternatively, the **Trek.R** file can be edited to set the desired arguments, and executed from the command line via R CMD BATCH or Rscript. Trek also features a browser-based graphical user interface (GUI) that can be executed locally on as many CPUs as desired. To launch the GUI, enter the **TrekGUI/** subfolder and double click on **TrekGUI** file. If the file fails to open a browser, make sure that the permissions are correctly set for the file (as executable):

```
$ cd TrekGUI/
$ chmod 700 TrekGUI # May require sudo rights.
```

If double clicking on **TrekGUI** does not launch your browser after the above step, then, most probably, the Rscript utility of R is not installed on the default */usr/bin/Rscript* path. To correct the TrekGUI setup, first find out the installed path for Rscript by typing from the command line:

```
$ which Rscript
```

then open the **TrekGUI** executable file via a usual plain text editor and correct the Rscript path stated at the first line. As soon as double clicking on **TrekGUI** opens the browser, the rest is self explanatory.

L1 type	N^{hg}	age, myr
L1Hs	1528	3.1
L1PA2	4867	7.6
L1PA3	10565	12.5
L1PA4	11763	18.0
L1PA5	11171	20.4

Table S1. Number of genomic copies and estimated age of hominoid L1 subfamilies. The numbers of L1 mobile elements (N^{hg}) in the human genome were revealed through the RepeatMasker [1] processing of the genome. The divergence age estimation was obtained from the published molecular clock analysis [2].

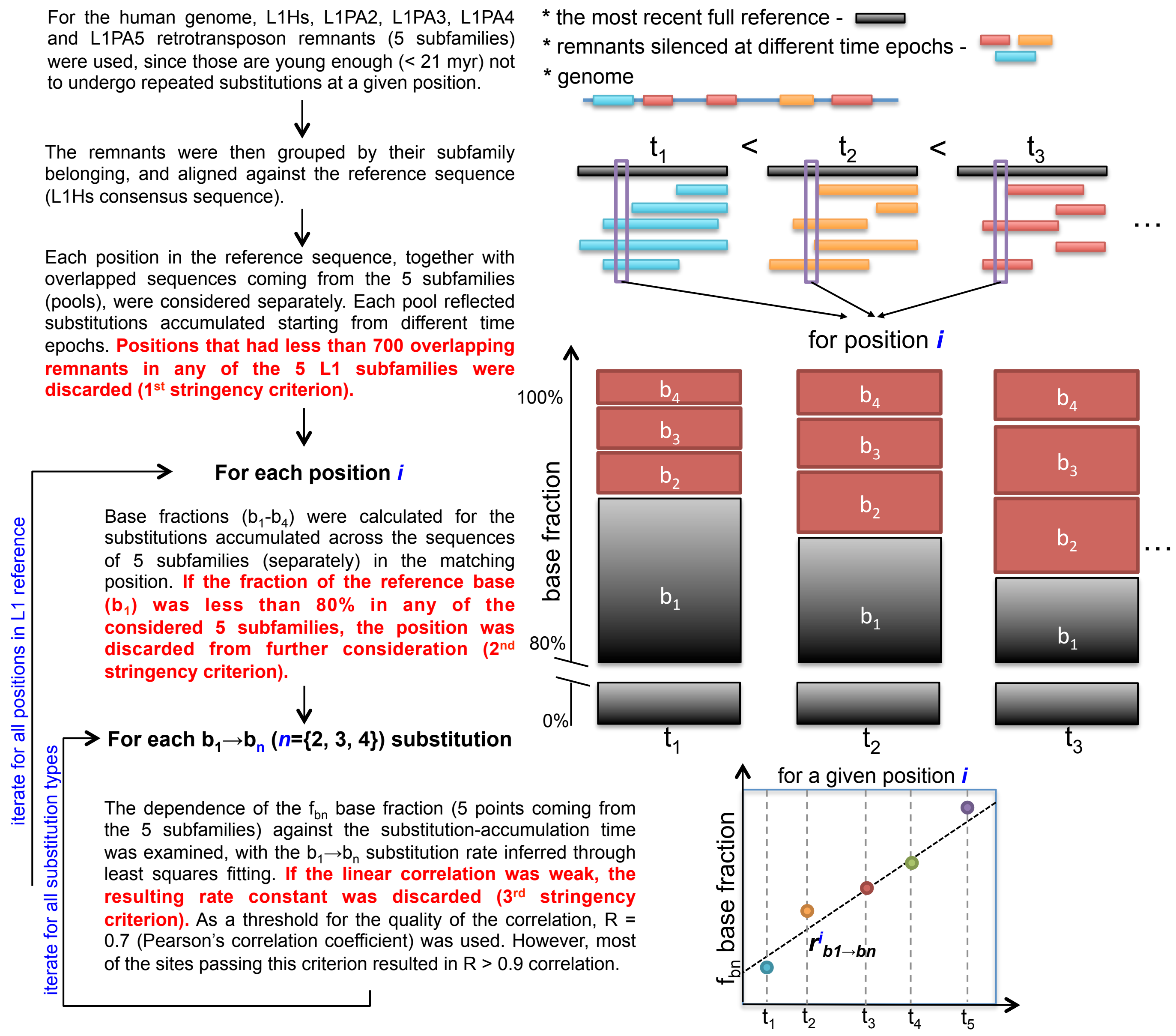

Figure S1. The used procedure of determining the context-dependent substitution rate constants. The applied stringency criteria are highlighted in red, with further details brought in **Methods**.

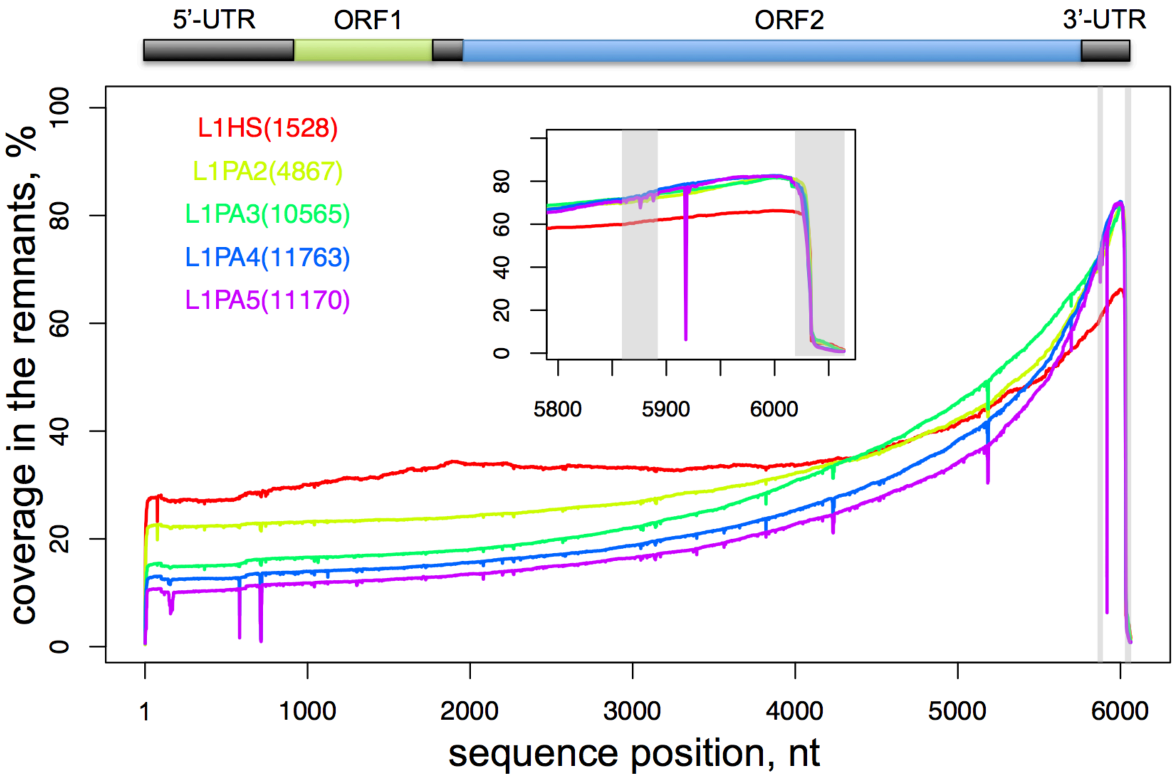

Figure S2. Position-wise coverage of the five subfamilies of L1 remnants in the human genome. All retrotransposon types, which are not too ancient for the time-accumulated substitutions to severely decrease the information content, were pairwise aligned on the 6064-nt consensus sequence of the human-specific L1Hs subfamily (reference sequence). The graph shows the percentage of cases where, for each considered L1 subfamily, the remnant sequences were mapped onto the corresponding position (x-axis). The gene organisation in these L1 elements is displayed on top, highlighting the terminal untranslated regions, along with two open reading frames (ORF1 and ORF2) and the short inter-ORF region. The positions 5856-5895 and 6018-6064, close to the 3'-end (also zoomed in the sub-plot) that engulf the low-complexity G-rich and A-rich sequences are marked with grey bands and excluded from the substitution rate analyses. The colour coding of the examined hominoid L1 retrotransposons, along with the genomic copy numbers in the human genome, is shown on the plot. The abrupt drops in the coverage at different positions along the sequence are because of deletions. The figure highlights the incomplete 5'-reverse transcription, characteristic to LINE elements. The poly-A tail at the 3'-end is also largely incomplete in most L1 remnants. It is interesting to note that the further back in time we go in terms of the activity period of the L1 subfamily, the less preserved the 5'-side of the L1 elements become relative to the 3'-end. An overall decrease of the preservation, hence coverage, is expected for the more ancient subfamilies, but the observed decrease relative to the 3'-end can indicate that the more recent subfamilies evolved a more efficient retrotransposition and/or improved transposonic RNA stability that results in more complete insertions.

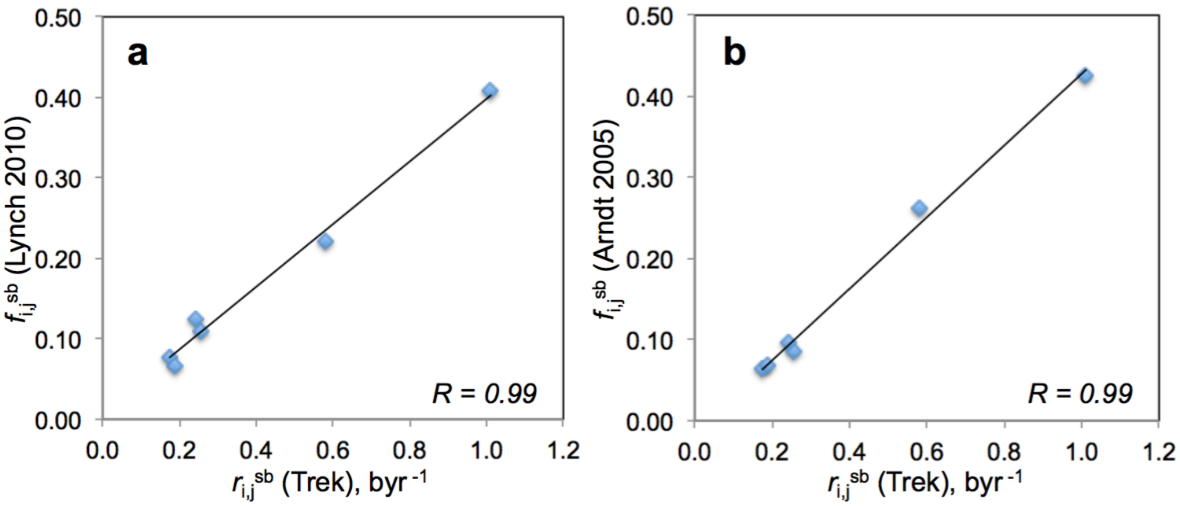

c

substitution type	Trek (this work) $r_{i,j}^{sb}$, byr^{-1}	Lynch (2010) $f_{i,j}^{sb}$	Arndt (2005) $f_{i,j}^{sb}$
A:T to C:G	0.175	0.078	0.064
A:T to G:C	0.582	0.221	0.262
A:T to T:A	0.188	0.067	0.068
G:C to T:A	0.256	0.110	0.085
G:C to C:G	0.243	0.125	0.095
G:C to A:T	1.011	0.408	0.425

Figure S3. Demonstration of the unbiased averaging of the substitution rates in the Trek procedure. (**a** and **b**) The median values of the Trek-reported $r_{i,j}^{core} = r_{i,j}^{sb} + \delta r_{i,j}^{sr}$ substitution rates, which should be reasonable estimates of single-base genomic average $r_{i,j}^{sb}$ rates (in time domain), are compared with two published datasets [3,4] for $r_{i,j}^{sb}$, expressed by normalised substitution fractions. Since both datasets report on strand-symmetry-accounted six unique rates, we have performed the same strand-symmetry averaging of the median values shown in **Figure 3** before the comparison. The Pearson's correlation coefficients are shown on the plots **a** and **b**. (**c**) The underlying numerical data.

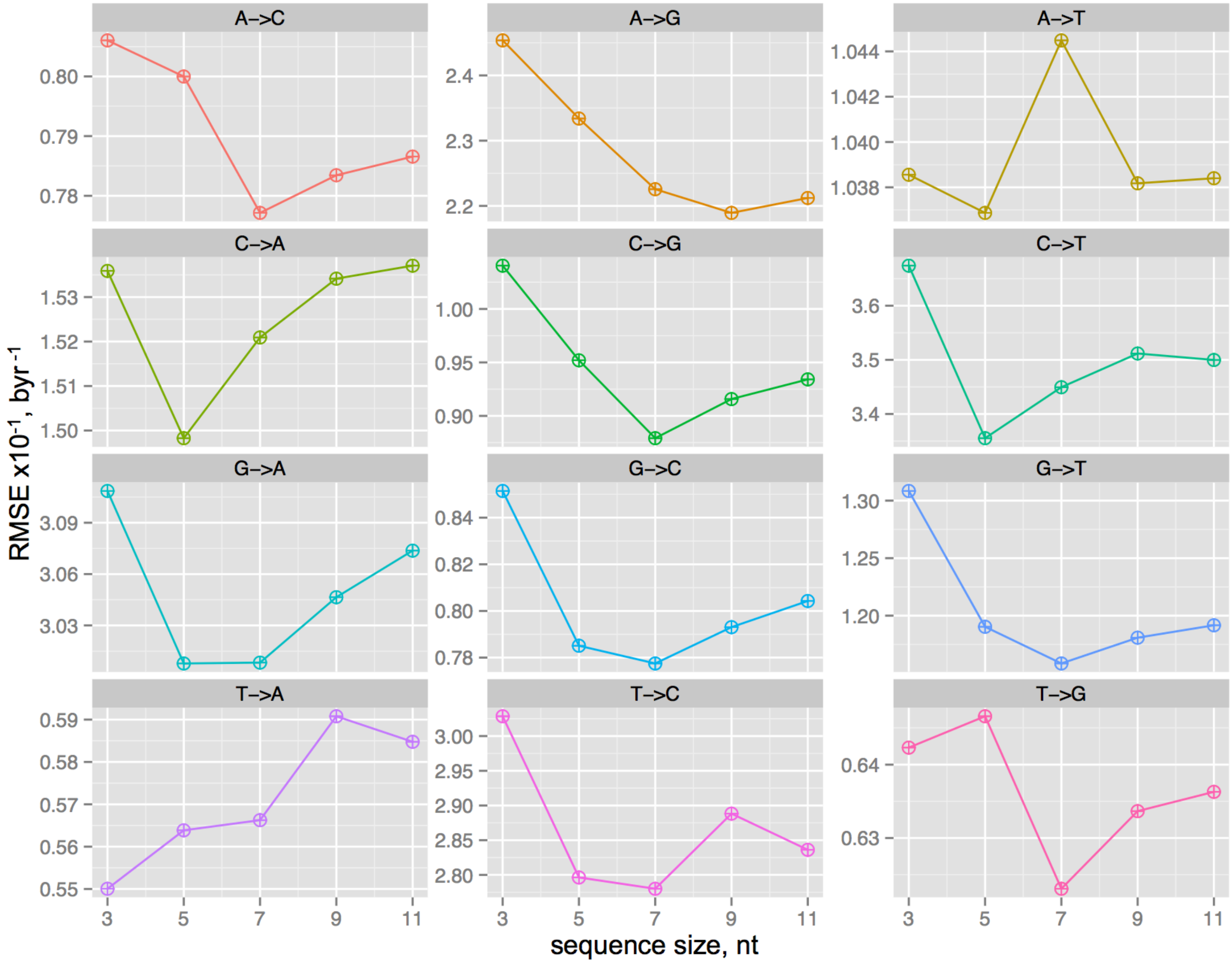

Figure S4. Selection of the optimal short-range sequence length directly influencing the substitution rates. The root-mean-squared errors of the rate constant prediction from only the neighbouring base information is presented as a function of the accounted sequence length, where the substitutions occur at the central base. The examined lengths are thus odd numbers, to allow equal number of upstream and downstream bases around the substitution point. The test models for the rates, to assess the optimal sequence length, were built using the machine learning, generalised boosted models. The 5- and 7-nt sequence lengths are found to be the best for general applicability for all the substitution types. Note that the machine learning predictions here are solely for finding out the optimal sequence length and, for the actual $r_{i,j}^{core}$ determination, a direct model-free mapping to the Trek substitution database is used.

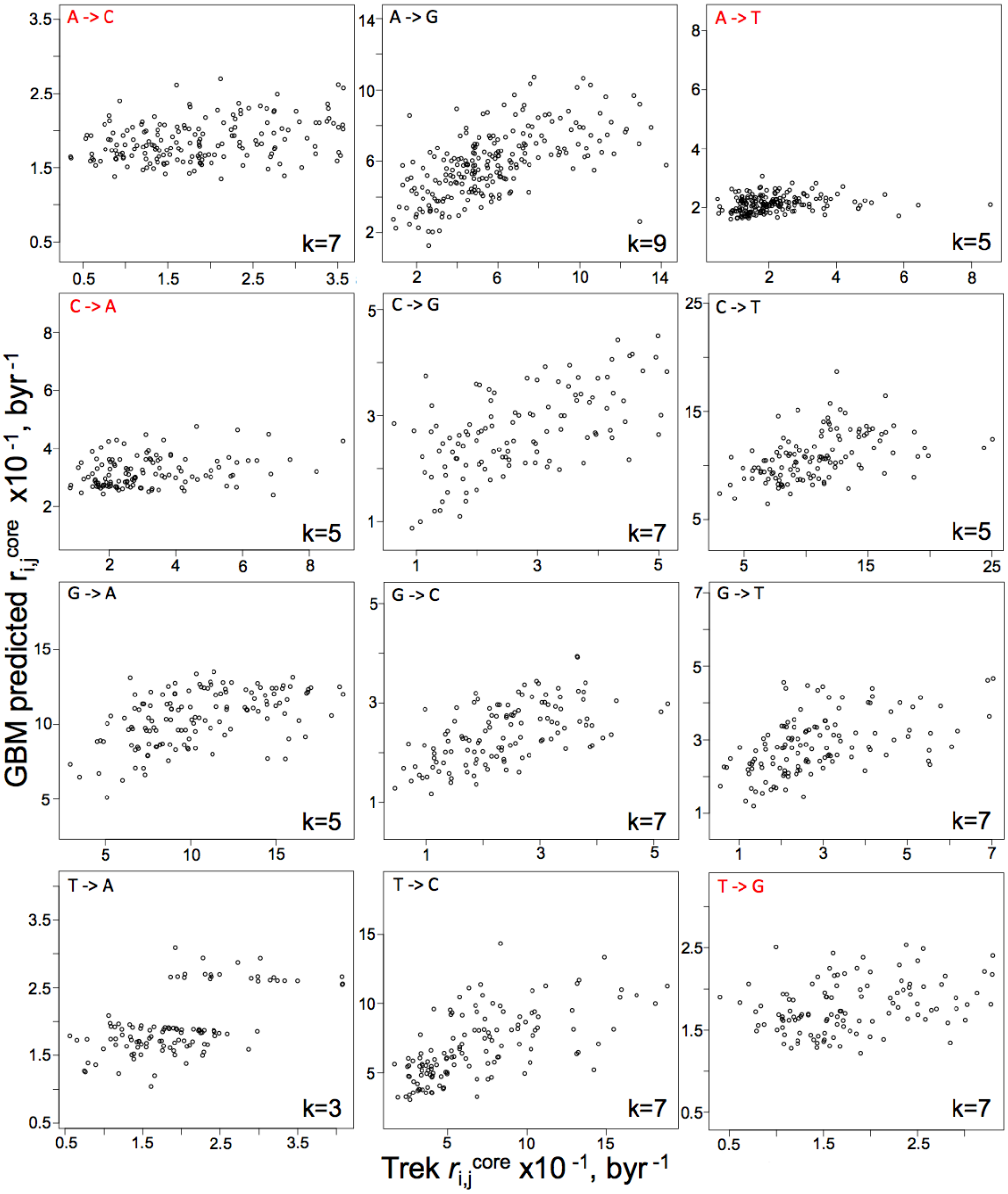

Figure S5. Performance of the optimal GBM models in the test constructs. Machine learning models were built to predict the $r_{i,j}^{core}$ constants using only the knowledge of the neighbouring bases. The resulting highest performing lengths of the sequences (k-mers) are shown at the bottom right corners of each plot. The best models identified for all $i \rightarrow j$ substitution types are presented here as an example of the predictability of the substitution rates from the neighbouring residues. We have used these analyses to infer the optimal sequence (k-mer) length, found to be around 5-7 nt. We then utilised the found maximum length (7 nt) for all the substitution types as a factor to stratify the Trek $r_{i,j}^{core}$ constants for the direct mapping to any given sequence.

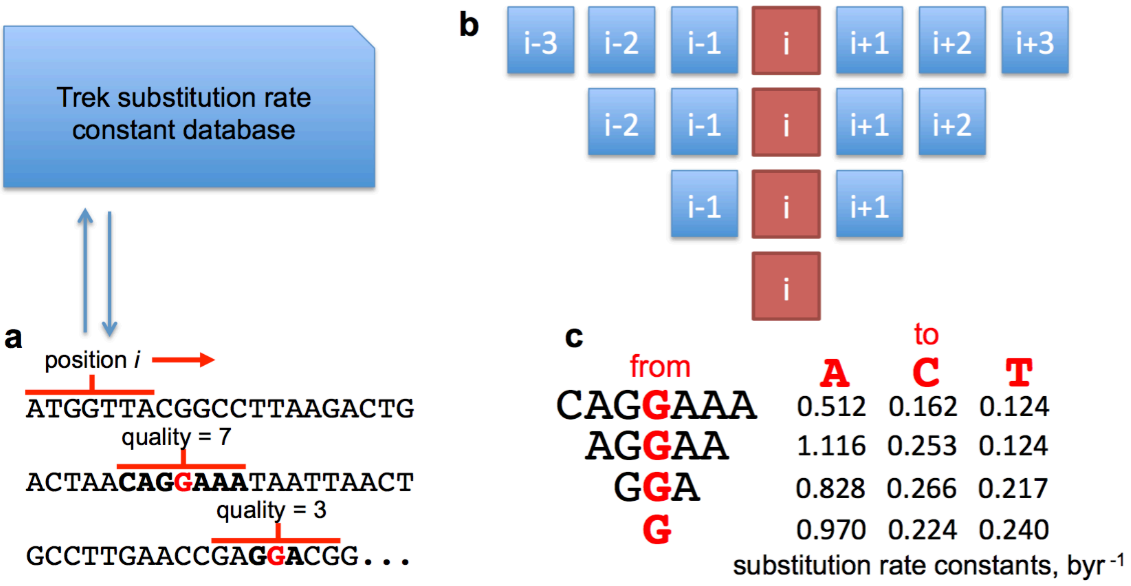

Figure S6. Procedure of mapping the $r_{i,j}^{core}$ constants onto any sequence. (**a**) A query sequence is analysed by examining each base via a 7-nt window centred at the position of the base. To reveal the rate constants for the three substitutions of the central base into the three other bases, the Trek database is searched. The Trek data contain information on the full set of unique k-mers (k = 1, 3, 5 and 7) found in the reference L1 element, along with their respective three substitution rate constants. The Trek data are averaged where multiple values are found because of multiple k-mer copies in the same L1 reference sequence. (**b**) In case a representative match is not found with the full 7-nt long sequence, the window around the given position in a query sequence is shortened into the longest variant (5-nt, 3-nt or 1-nt) with a match detected in the Trek database. (**c**) A case for the average substitution rate constants of the base G converting to A, C or T, with different extent of context information in the Trek data.

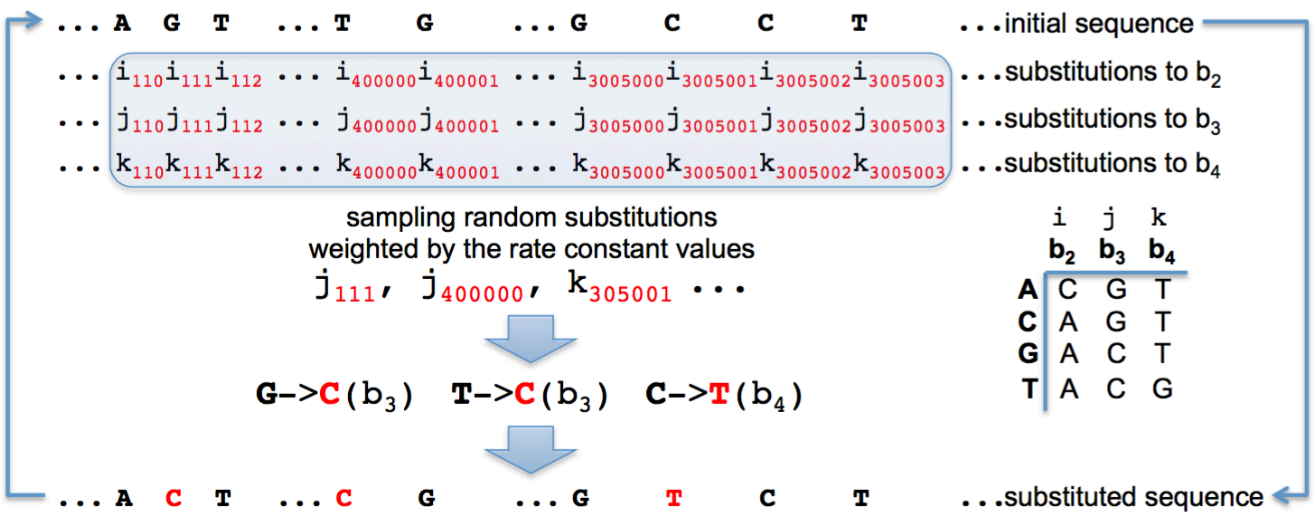

Figure S7. ***In silico*** **"evolution" of a genome with only $r_{i,j}^{core}$ substitution rate constants.** A random sequence of 5-mln-nt is generated with 60% G+C content (30% G, 30% C, 20% A, 20% T). Next, for each position, *pos*, the i_{pos}, j_{pos} and k_{pos} rate constants for the substitutions into the other three b_2, b_3 and b_4 bases are obtained from the Trek database, using the 7-nt sequence window centred at each *pos*. This generates 15-mln (3 times the sequence size) rate constant values that, for the first-order kinetic processes, are proportional to the substitution probabilities. We then sampled 5000 instances from the 15 mln generated set of *i*, *j* and *k*, with the sampling process weighted by the i_{pos}, j_{pos} and k_{pos} values. Those sampled instances contain information on both the substitution positions (*pos* subscript) and the substitution types (*i*, *j* and *k* describing the substitutions to b_2, b_3 and b_4 bases respectively, the latter ones being bases other than the initial base, always ordered alphabetically). After performing the sampled substitutions, we repeat the cycle and continue the process each time with substitution rates updated wherever the already performed substitutions affect the values. We continue the simulation until the equilibration of the sequence composition.

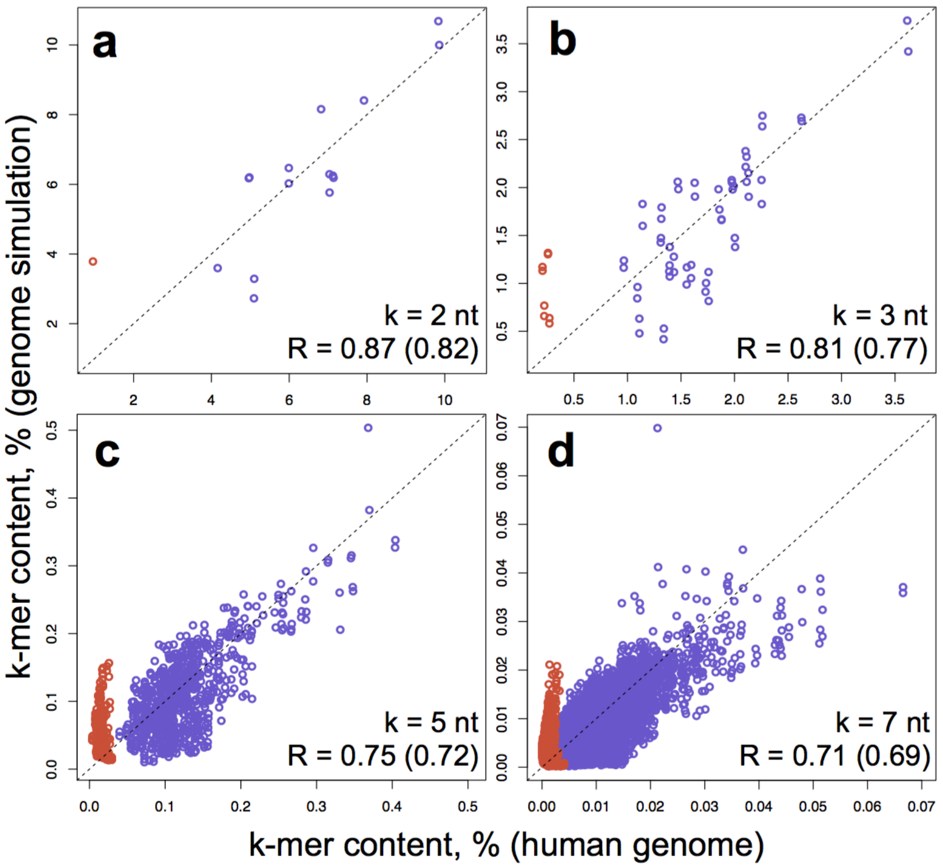

Figure S8. Comparison of the *in silico* (no strand-symmetries) evolved and real human genomes. (**a**-**d**) The *in silico* genome is equilibrated here by using the raw Trek database but without accounting for the strand-symmetries of the $r_{i,j}^{core}$ substitution rates (contrasting with **Figure 4d**-**g**). The plots **a**-**d** show the correlation of different k-mer contents in the equilibrated sequence with the corresponding content in the real human genome. The lengths of the k-mers along with the correlation coefficients are shown on the bottom right corners of the figure. Two correlation coefficients are shown with the exclusion and the inclusion (the value in the bracket) of the CpG containing oligomers (red points in the plots). The dashed lines depict the diagonals for the ideal match of the k-mer contents.

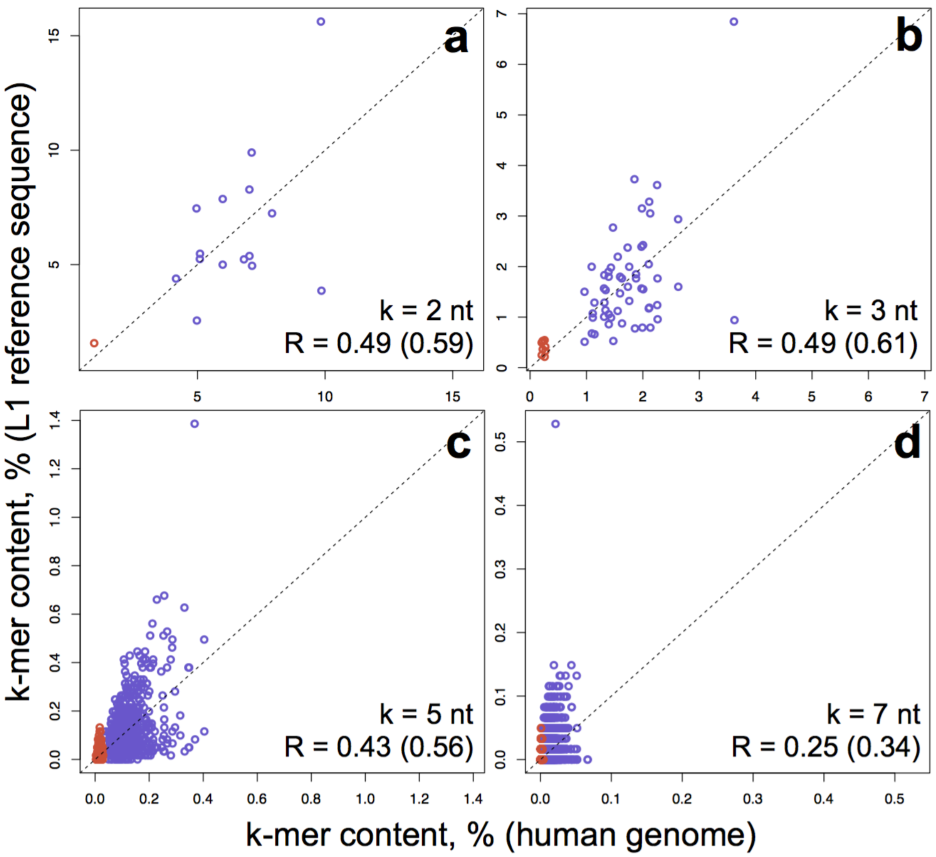

Figure S9. Oligomeric composition of the L1 reference sequence compared to that of the human genome. (**a-d**) The correlation plots between the oligomeric composition in the used L1 reference sequence (human L1Hs consensus sequence, y-axis) and the human genome (x-axis) for different (k = 2, 3, 5, 7) k-mers. The red data points are from CpG-containing k-mers. The analysis shows that the 6064-nt-long L1 elements are rather different from the human genome in their k-mer contents. This observation, besides the specific ways by which we extracted the neutral core substitution rate constants for the human genome, outlines the absence of a direct circularity in the reproduction of the human genome k-mer contents through the Trek $r_{i,j}^{core}$ constants (**Figure 4d-g**).

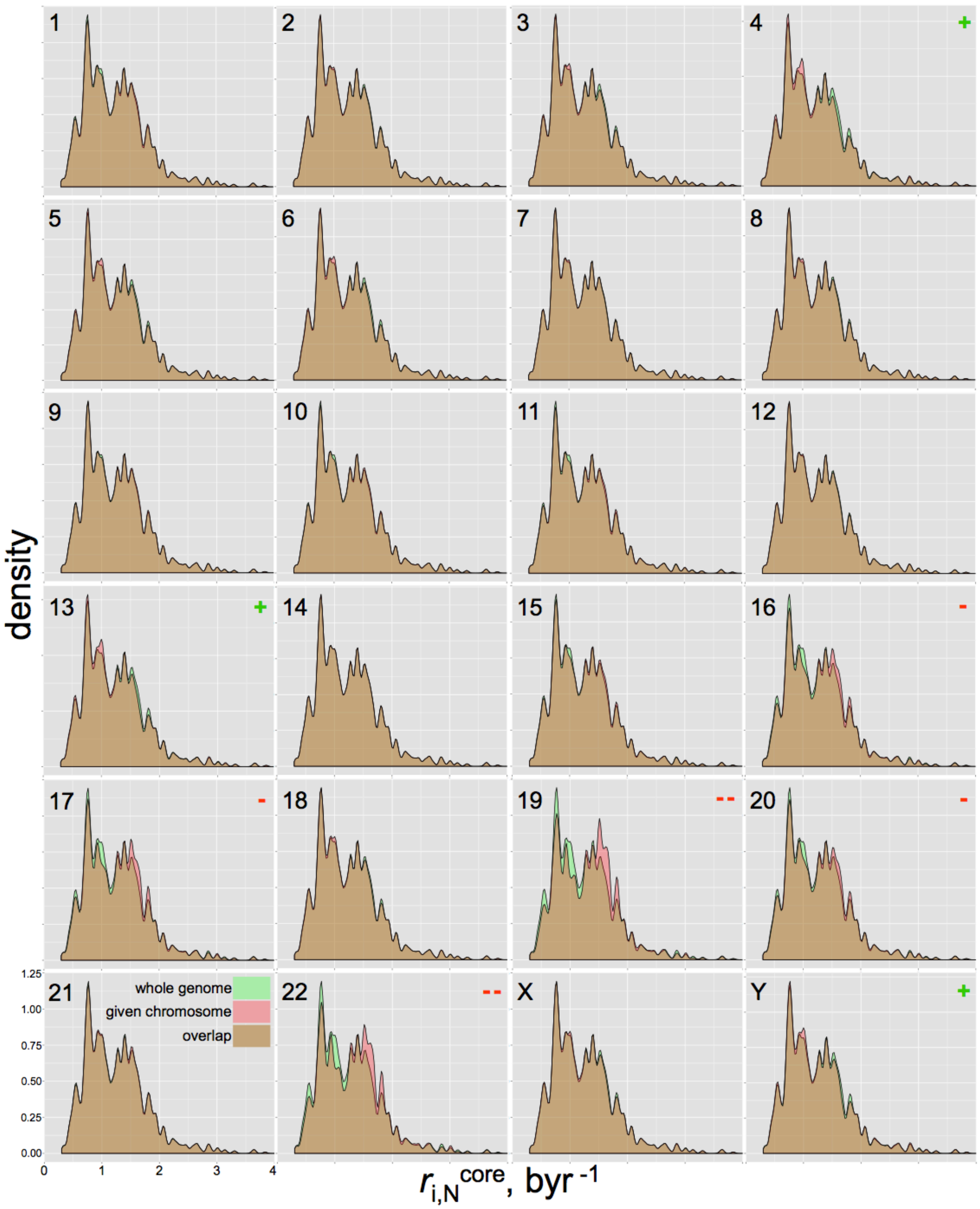

Figure S10. Basal substitution propensity (BSP) profiles of the human genome and its individual chromosomes. The plots show the density (kernel density estimate) distribution of the BSPs in the whole human genome (green), as compared to the individual chromosomes (red). The chromosome types are shown at the top-left corners of the plots. The overlaps of both distributions are in brown. The x-axis shows the BSP for the substitution to any other base ($r_{i,N}^{core} = r_{i,b2}^{core} + r_{i,b3}^{core} + r_{i,b4}^{core}$), as inferred from mapping the positions with the context information (up to 7-mers) to the Trek database. Most of the chromosomes repeat the whole-genome substitution profile, with the significant exceptions noted for the chromosomes 19 and 22 that are relatively "destabilised", in part due to their high G+C contents. The type (+ for stabilisation and - for destabilisation) of the difference between the profiles are shown at the top-right corners of the plots.

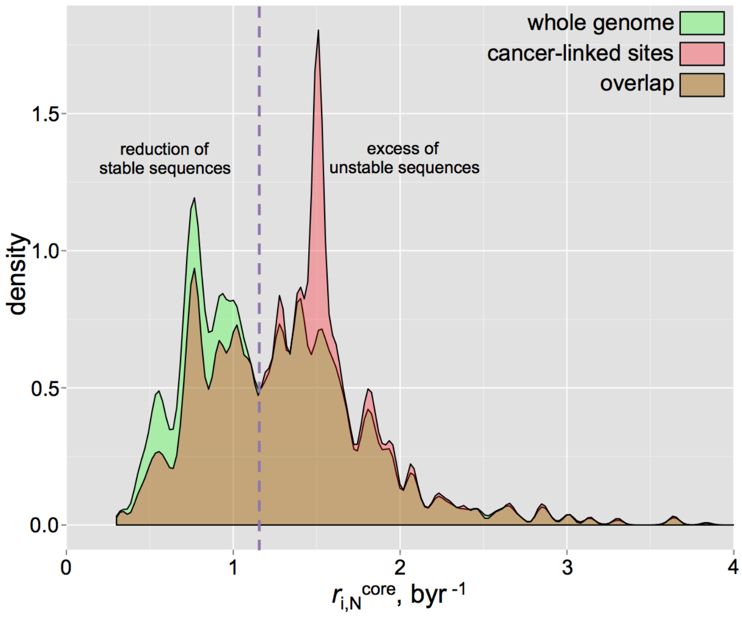

Figure S11. Basal substitution propensity (BSP) profiles of the human genome and cancer-linked somatic mutation sites. The density (kernel density estimate) distribution of the BSPs in the whole human genome (green), compared to the sites of the mutations associated with cancer (red) are shown. The overlaps of both distributions are in brown. The x-axis shows the BSP for the substitution to any other base ($r_{i,N}^{core} = r_{i,b2}^{core} + r_{i,b3}^{core} + r_{i,b4}^{core}$). The comparison shows a relative reduction of the stable sites and excess of the unstable sites in cancer-linked loci.

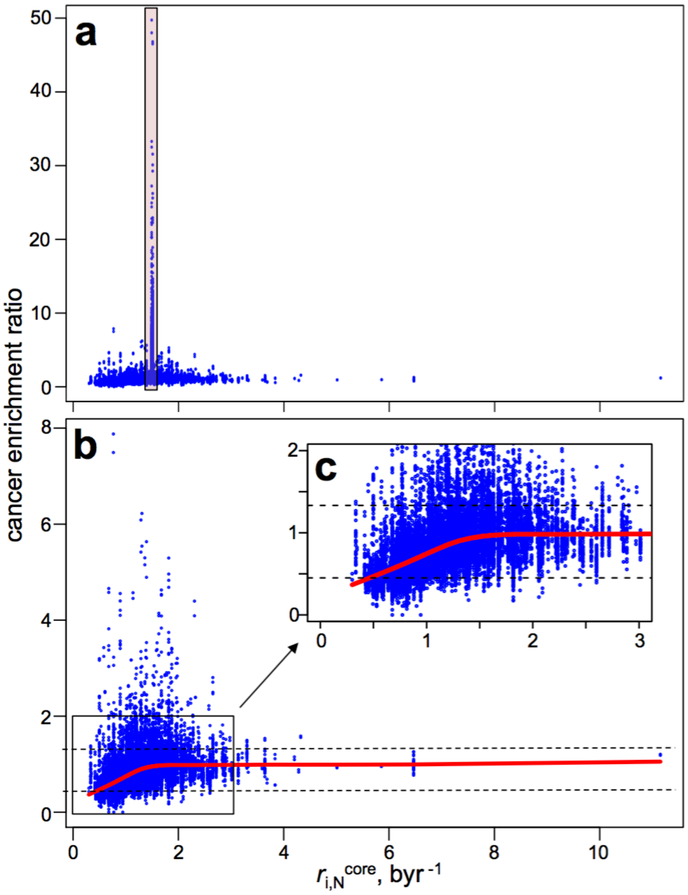

Figure S12. Enrichment of 7-mers with varying BSPs in the cancer-linked somatic mutation sites. (**a**) The 4^7 points in the plots correspond to unique 7-mer sequences. The cancer enrichment score for each such sequence was calculated by dividing the occurrence fraction of the sequence in only the cancer-linked sites to the fraction in the whole repeat-masked human genome. All the 7-mers that had either C or G of a CpG dyad at the centre show a remarkable cancer enrichment ratio (up to 49, see the points in the red box in A). Since for the CpGs, Trek data, by design, report on only the average C and G substitution rate constants, the BSP values for those points can be underestimated in **a**. However, even the average BSPs for C and G bases are higher than the discussed 1.13 byr^{-1} threshold. Accounting for the epigenetic methylation-driven increase in BSPs for the CpG containing sequences would only increase their BSP values. (**b** and **c**) The plots represent the data that exclude the sequences with CpGs at the centre. The mean cancer enrichment ratio for such subset was 0.89, with standard deviation of 0.44. The data points within the 0.89±0.44 range of cancer enrichment ratio are contained in between the dashed lines in **b** and **c**. The red lines in **b** and **c** represent the Lowess [5] fit, showing the decrease of the cancer enrichment ratio with the decrease in BSP.

Description of the Additional file 2

Additional_File_2.txt

data_1

Data on the Trek $r_{i,j}^{core}$ core substitution rate constants in the L1 reference sequence (L1Hs). The columns hold the substitution types, sequence positions, sequence contexts with 5 upstream and 5 downstream bases, $r_{i,j}^{core}$ constants (byr^{-1}), the correlation coefficient of the linear fit behind the $r_{i,j}^{core}$ inference and the significance t-values.

data_2

Trek database processed with strand-symmetry consideration. The file contains all 7-mers, outlining the central base (*i*) where the rate constants (byr^{-1}) are given for the core neutral substitutions into the 3 non-*i* bases (shown in an alphabetical order). The final columns hold the quality scores for $r_{i,j}^{core}$ (actual sequence lengths matched with L1).

data_3

Trek database processed without strand-symmetry consideration. The file contains all 7-mers, outlining the central base (*i*) where the rate constants (byr^{-1}) are given for the core neutral substitutions into the 3 non-*i* bases (shown in an alphabetical order). The final columns hold the quality scores for $r_{i,j}^{core}$ (actual sequence lengths matched with L1).

data_4

k-mer content for the repeat masked and unmasked versions of the human genome (RefSeq, hg19/GRCh37). The text file contains sections with headers showing the k-mer size and the genome masking status.

data_5

The full set of all 7-mer sequences with the respective cancer enrichment scores and basal substitution propensities. These data are based on the comparison of the COSMIC dataset with the 7-mer distribution in the human RefSeq. Only the non-SNP and non-coding sites undergoing single-nucleotide substitutions are taken from COSMIC (NCV set).

data_6

The GBM parameters that minimise the error of the test tree-based models, used to infer the optimal length window of neighbour effects. The columns represent the found best tuning parameters, along with the RMSE values for each $i \rightarrow j$ substitution type and window length.

Supplementary references

1. Smit AFA, Hubley R, Green P. *RepeatMasker Open-4.0*. 2015. Available from: http://www.repeatmasker.org

2. Khan H. Molecular evolution and tempo of amplification of human LINE-1 retrotransposons since the origin of primates. Genome Res. 2006;16:78–87.

3. Arndt PF, Hwa T, Petrov DA. Substantial regional variation in substitution rates in the human genome: importance of GC content, gene density, and telomere-specific effects. J. Mol. Evol. 2005;60:748–63.

4. Lynch M. Rate, molecular spectrum, and consequences of human mutation. Proc. Natl. Acad. Sci. USA. 2010;107:961–8.

5. Cleveland WS. Robust locally weighted regression and smoothing scatterplots. J. Am. Stat. Assoc. 1979;74:829–36.
